# Turning Cold Tumors Into Hot Tumors: Implications for Cancer Therapy

**DOI:** 10.1002/mco2.70882

**Published:** 2026-08-03

**Authors:** Xiao Ge, Dong Han, Jing Wang, Xinyu Dai, Chen Ma, Feihong Chen

**Affiliations:** ^1^ Department of Chemical Biology and Pharmaceutical Engineering, School of Chemistry and Chemical Engineering Southeast University Nanjing China; ^2^ Department of Biomedical Engineering, College of Engineering and Applied Sciences, State Key Laboratory of Analytical Chemistry for Life Science Nanjing University Nanjing China

**Keywords:** cold‐to‐hot tumor conversion, immune evasion, immunotherapy resistance, immunotherapy resistance plasticity, tumor microenvironment

## Abstract

The prevailing view of cold tumors as a singular immune‐insensitive state is not merely imprecise, which is clinically misleading. Currently, we contend that cold tumors comprise a heterogeneous spectrum of resistance ecosystems by distinct, coexisting, dominant barriers. We argue that a universal “heating” strategy is unlikely to succeed; instead, durable clinical benefit will depend on barrier‐directed immune reprogramming. We dissect this heterogeneity by linking a set of core mechanistic drivers to canonical hot, immune‑desert, and immune‐excluded phenotypes. The drivers include defective antigen processing and presentation, impaired T‐cell priming, physical exclusion by aberrant vasculature, stromal components, metabolic exhaustion, and adaptive resistance. Our evaluation of emerging conversion strategies focuses not on their novelty but on capacity to neutralize a specific rate‐limiting barrier. We also examine why combination and sequencing are indispensable, highlight translational gaps between preclinical models and human disease and propose a dynamic biomarker framework that extends beyond static PD‐L1 assessment to capture real‐time shifts in dominant barrier. Our central conclusion is that durable cold‐to‐hot conversion will require iterative, biomarker‐guided and barrier‐adaptive interventions that evolve alongside tumor counter‐adaptation. The reframing positions immunotherapy resistance not as a terminal obstacle but as a manageable, dynamic challenge, offering a practical strategy for clinical translation.

## Graphical abstract

Barrier‐defined strategies for cold‐to‐hot tumor conversion. Cold tumors include immune‐desert and immune‐excluded states marked by poor antigen visibility, limited T‐cell priming, stromal or vascular exclusion, metabolic stress and suppressive immune programs. Barrier‐matched interventions can restore tumor visibility, enable immune‐cell access, preserve T‐cell fitness and reprogram the tumor microenvironment. These coordinated strategies promote intratumoral CD8^+^ T‐cell and NK‐cell infiltration, dendritic‐cell maturation, antigen presentation, vascular normalization and tumor‐cell killing, thereby supporting the formation of an inflamed, accessible, and immune‐responsive tumor microenvironment. Precision implementation requires barrier‐defined profiling, biomarker‐guided monitoring, longitudinal assessment and adaptive combination design. CAF, cancer‐associated fibroblast; ECM, extracellular matrix; IFN, interferon; MDSC, myeloid‐derived suppressor cell; NK, natural killer; TAM, tumor‐associated macrophage; TME, tumor microenvironment; Treg, regulatory T cell.

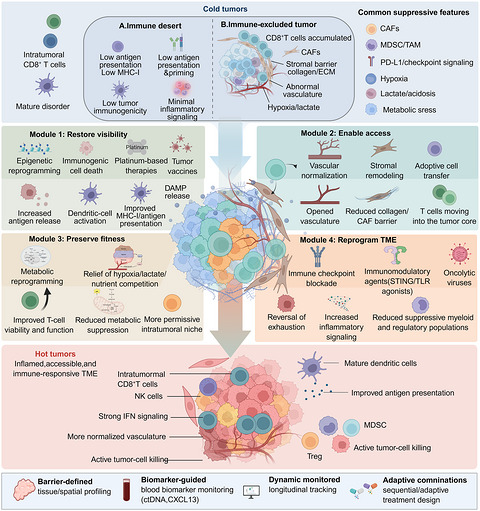

## 1 Introduction

Cancer immunotherapy can durably control tumors by engaging host immunity [1, 2]. Checkpoint blockade, adoptive cell transfer (ACT), therapeutic cancer vaccines, and related immune‐based modalities produce sustained responses in subsets of patients across multiple tumor types [3], yet primary resistance and relapse remain frequent [4]. These observations point to a key determinant: therapeutic efficacy depends not only on the drug and its target, but also on the immune landscape of the tumor microenvironment [5, 6]. Although heuristic, the hot–cold tumor classification underscores the immune heterogeneity that underlies divergent clinical outcomes.

Hot tumors typically display an inflamed microenvironment, with substantial effector T‑cell infiltration, active interferon signaling, and ongoing antigen presentation and immune recognition [7, 8]. Although these tumors often express inhibitory checkpoints, they retain endogenous antitumor immunity that can be therapeutically reactivated [9, 10]. Cold tumors, by contrast, are defined by limited T‑cell infiltration, low intrinsic immunogenicity, defective antigen processing and presentation, impaired dendritic‑cell priming, constrained immune‑cell trafficking, or dominant local suppression [10, 11]. They are not a single entity but encompass at least two noninflamed states: immune‑desert tumors, in which effective priming is absent; and immune‑excluded tumors, in which immune cells accumulate at the tumor margin or within stroma without penetrating tumor nests [7, 8]. This phenotypic spectrum arises from tumor‑intrinsic and tumor‑extrinsic mechanisms alike. Low neoantigen burden, oncogenic signaling, defective MHC presentation, abnormal vasculature, extracellular matrix remodeling, cancer‑associated fibroblasts, suppressive myeloid cells and regulatory T cells, hypoxia, and metabolic competition within the tumor microenvironment all contribute to this state [12, 13]. Tumor coldness therefore represents an actively enforced state of immune evasion, not a passive lack of inflammation.

This distinction carries direct therapeutic weight. Approved immunotherapies generally achieve greater efficacy in tumors with pre‑existing immune engagement [14, 15]. Noninflamed tumors, in contrast, typically require active reprogramming before checkpoint blockade or other T‑cell‑dependent strategies can yield meaningful responses [16, 17]. A core therapeutic challenge therefore extends beyond relieving inhibitory checkpoints; it also demands restoration of the upstream processes that enable productive antitumor immunity, including immunogenic cell death (ICD), antigen release, dendritic‑cell activation, T‑cell priming, immune‑cell trafficking, intratumoral infiltration, and sustained effector function within the microenvironment [18, 19, 20].

Multiple modalities are being explored to facilitate this conversion, including immune checkpoint inhibitors, therapeutic vaccines, ACT, oncolytic viruses, immunomodulatory agents, epigenetic modifiers, and rational combinations [21, 22]. Conventional anticancer treatments may also contribute under specific conditions. Platinum‑based regimens, for instance, remain broadly used across many solid tumors and, in appropriate settings, can promote ICD, enhance tumor antigenicity, remodel the microenvironment, and augment immunotherapy sensitivity [23, 24]. Durable cold‑to‑hot conversion, however, remains elusive. Major obstacles include marked interpatient and intratumoral heterogeneity, temporal plasticity of immune phenotypes, adaptive resistance, treatment‑related toxicities, and a persistent lack of robust biomarkers for patient selection and therapeutic stratification [8, 25].

This review examines the biological and clinical foundations of hot and cold tumor states, alongside current barriers to effective immunotherapy. We discuss key immune evasion mechanisms and the microenvironmental determinants of immune‑desert and immune‑excluded phenotypes. We then survey established and emerging conversion strategies, their impact on immune remodeling, and the translational evidence supporting them. We also address preclinical models, predictive biomarker frameworks, safety considerations, and future directions aimed at broadening both the reach and durability of immunotherapeutic responses.

## 2 Mechanisms of Tumor Immune Evasion

Immune evasion enables malignant cells to escape immune recognition and elimination. This process is fundamental to cold tumor formation and represents a primary obstacle to effective immunotherapy [26, 27]. Evasion is not a unitary defect but a multilayered phenomenon. It encompasses reduced tumor immunogenicity, impaired antigen presentation and T‐cell priming, checkpoint‑mediated suppression of lymphocyte function, and immune‑driven evolutionary diversification. These interrelated mechanisms collectively attenuate antitumor immunity and establish the biological groundwork for immune‑cold disease.

### 2.1 Genetic and Epigenetic Determinants of Immune Escape

Genetic and epigenetic alterations govern baseline immunogenicity and establish immune‑resistant states, positioning them as upstream drivers of immune evasion [28, 29]. Oncogenic activation of KRAS, PIK3CA and MYC suppresses antigen presentation, upregulates immune checkpoints and promotes immunosuppressive cytokine production [30, 31]. KRAS activation, for instance, enhances CD47 expression and impairs phagocytosis, whereas the PIK3CA H1047R mutation reduces CD8^+^ T‐cell infiltration and expands inhibitory myeloid populations [30, 32]. Loss of tumor suppressors further reinforces evasion. TP53 loss diminishes MHC Class I expression and thereby restricts recognition by cytotoxic T cells, whereas PTEN deficiency activates PI3K–AKT signaling and drives PD‑L1 expression [33, 34, 35, 36].

Epigenetic reprogramming adds another layer of immune regulation. Hypermethylation of promoters governing MHC Class I genes and antigen‑processing components attenuates antigen presentation [37, 38]. Histone modifiers, including HDACs and EZH2, repress immune‑related genes via chromatin remodeling [39, 40]. Noncoding RNAs such as miR‑21, lncRNA NEAT1, and circRNA‑002178 further modulate PD‑L1 expression and cGAS–STING signaling [41, 42, 43]. Together, these alterations reduce tumor visibility to the immune system and create a molecular substrate for downstream immune dysfunction.

### 2.2 Defects in Antigen Presentation and T‐Cell Priming

Defective antigen presentation and T‐cell priming represent a second major hurdle for antitumor immunity [44, 45]. At the tumor‑cell level, downregulation or loss of MHC Class I impairs presentation of tumor‑specific antigens to CD8^+^ T cells. This defect is often compounded by abnormalities in the antigen‑processing machinery, including LMP2, LMP7, and ERAP1, which further compromise peptide loading and presentation [46, 47, 48]. In parallel, immunoediting selects tumor subclones with reduced neoantigenicity, facilitating escape from immune surveillance [37, 49].

Effective priming is also constrained by dysfunction within the dendritic‐cell compartment. TIM3‑mediated inhibition of extracellular DNA sensing and disruption of cGAS–STING signaling impair dendritic‐cell maturation and weaken T‐cell priming [50]. Downregulation of co‑stimulatory molecules such as CD80 and CD86, together with persistent tumor‑derived inhibitory signals, further compromises naïve T‐cell activation [51, 52]. These defects collectively deprive the immune system of both sufficient antigenic targets and adequate antigen‑presenting capacity, thereby promoting the establishment of immune‑cold tumors.

### 2.3 Immune Checkpoint‐Mediated Suppression of Antitumor Responses

Even when tumor‑specific lymphocytes are generated, their effector functions can be restrained by immune checkpoint signaling [53, 54]. The PD‑1–PD‑L1 axis is the most extensively studied example. PD‑L1 can be induced by interferon‑γ as part of adaptive resistance or by oncogenic pathways including PI3K–AKT signaling, leading to T‐cell exhaustion and functional impairment [55, 56, 57]. Additional checkpoint pathways also reinforce immune evasion. CTLA‑4 antagonizes CD28‑mediated co‑stimulation and supports regulatory T‐cell suppressive activity [58, 59]. LAG‑3, TIM‑3, and TIGIT further constrain T‐cell and NK‐cell function through distinct ligand interactions and are frequently co‑expressed on dysfunctional lymphocytes [60, 61, 62, 63]. CD47, in addition, suppresses macrophage‑mediated phagocytosis via SIRPα signaling [64, 65]. These pathways collectively establish a multilayered inhibitory network that enables tumors to sustain immune suppression even after immune recognition has occurred.

### 2.4 Tumor Heterogeneity and Adaptive Immune Escape

Tumor heterogeneity amplifies immune evasion and substantially limits durable therapeutic control [66, 67]. Intratumoral genetic and phenotypic diversity generates variable expression of neoantigens, MHC molecules and immune checkpoints, facilitating subclonal escape [68, 69]. Spatial heterogeneity produces regions with distinct immune accessibility and selective pressure, whereas temporal heterogeneity permits dynamic adaptation during tumor progression and treatment [66, 67, 68, 69]. Upregulation of alternative checkpoints such as TIM‑3 following PD‑1 blockade, for instance, can drive acquired resistance [68, 69, 70, 71]. Tumor heterogeneity thus operates not as an isolated mechanism but as an evolutionary framework that sustains immune escape and attenuates therapeutic efficacy.

**FIGURE 1:**
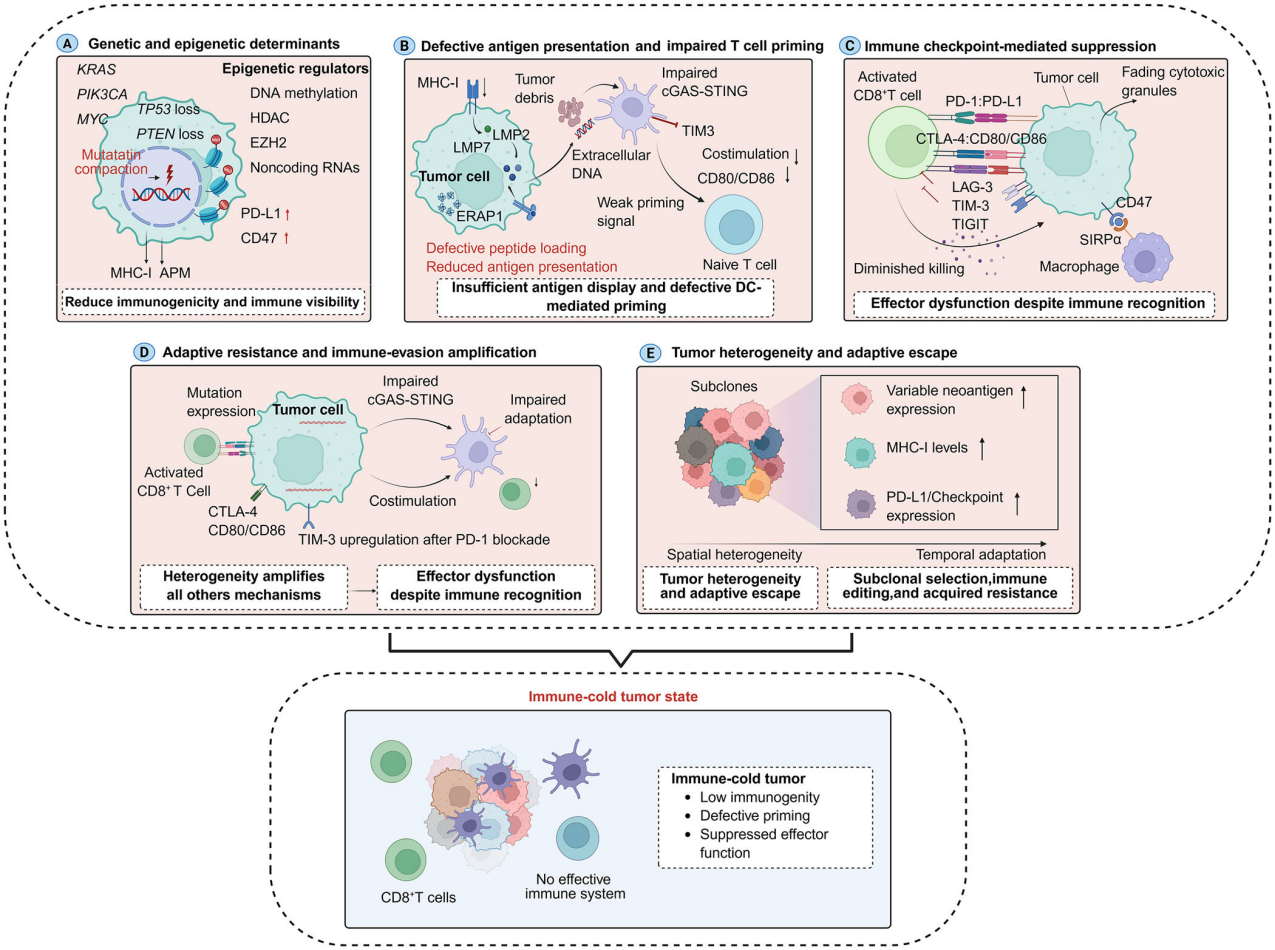
Mechanistic basis of immune evasion in immune‐cold tumors. Immune‐cold tumors are sustained by multiple, nonmutually exclusive mechanisms that reduce immune visibility, impair T‐cell priming and suppress effector function. The numbered panels indicate: (A) Genetic and epigenetic determinants of reduced tumor immunogenicity; (B) Defective MHC‐I antigen presentation and impaired dendritic‐cell‐mediated T‐cell priming; (C) Immune checkpoint‐mediated suppression of cytotoxic T cells and macrophage phagocytosis; (D) Adaptive resistance following immune pressure or checkpoint blockade; (E) Tumor heterogeneity‐driven immune escape through spatial and temporal subclonal evolution. Together, these mechanisms converge to establish an immune‐cold tumor state characterized by low immunogenicity, defective priming and suppressed effector function. APM, antigen‐processing machinery; DC, dendritic cell; MHC‐I, major histocompatibility complex Class I.

Tumor immune evasion involves mechanisms that lower immunogenicity, impair antigen presentation, and T‑cell priming, and dampen effector function after immune recognition. These mechanisms define the biological basis of immune‑cold tumors (Figure 1). Their maintenance, however, depends on the tumor microenvironment, where stromal, vascular, metabolic and immune components collectively reinforce immune suppression.

## 3 The Tumor Microenvironment: An Ecosystem That Stabilizes Cold Tumor States

The mechanisms described above do not operate in isolation. Within established tumors, they function within a microenvironment where stromal, vascular, immune and metabolic components interact to reinforce immune‑cold states [72, 73]. The tumor microenvironment thus constitutes more than a passive background; it represents an ecosystem in which defective priming, immune exclusion and suppressive signaling are spatially organized, amplified, and perpetuated. An ecosystem‑level view is essential to explaining the persistence of cold tumors and to understanding why effective intervention frequently demands barrier‑matched therapeutic strategies.

### 3.1 Composition, Heterogeneity, and Dynamic Evolution of the Tumor Microenvironment

The tumor microenvironment comprises a highly organized and dynamic array of immune cells, cancer‑associated fibroblasts, endothelial cells, extracellular matrix components, vascular networks, and soluble mediators [74, 75]. These constituents remodel continuously during tumor progression, metastasis, and treatment, with their functional states shaped by ongoing cellular crosstalk [75, 76, 77]. Heterogeneity manifests across patients, across lesions within the same patient and across disease stages [74, 78]. Systemic influences, including host metabolism, the gut microbiome, endocrine signaling, and circadian rhythms, further modulate local immune states [74]. Cold tumor formation thus arises not solely from tumor‑intrinsic programs but also from host physiology and dynamic ecological interactions.

### 3.2 Immune Landscape and Spatial Organization of Cold and Hot States

The tumor microenvironment can be assigned to distinct immune phenotypes based on the density, localization, and functional state of infiltrating immune cells [79]. Hot, or immune‑inflamed, tumors are rich in antigen‑presenting cells, activated CD8^+^ T cells, Th1 cells, and NK cells, alongside proinflammatory cytokines that sustain effective antitumor immunity [74, 79]. Cold tumors, in contrast, encompass at least two major spatial states. Immune‑desert tumors lack meaningful immune infiltration, whereas immune‑excluded tumors contain lymphocytes that remain confined to stromal regions or the invasive margin and fail to penetrate the tumor core [79]. This spatial distinction carries therapeutic relevance: immune‑desert tumors are more likely to require restoration of immune priming, whereas immune‑excluded tumors may necessitate disruption of stromal and vascular barriers to permit immune entry.

### 3.3 Immune Cell Remodeling in the Cold Tumor Microenvironment

Immune‐cell composition and function are remodeled in cold tumors, skewed toward suppression rather than immune surveillance [80, 81, 82]. Cytotoxic CD8^+^ T cells, NK cells, and NKT cells are frequently reduced in number or functionally exhausted, whereas professional antigen‑presenting cells, particularly conventional Type 1 dendritic cells (cDC1s), are often scarce or dysfunctional [74, 83, 84]. Given that cDC1s are indispensable for cross‑presentation and CD8^+^ T‐cell priming, their absence correlates closely with resistance to checkpoint blockade in preclinical models [50, 54, 85, 86, 87].

Concurrently, suppressive immune populations expand and dominate the local landscape. Regulatory T cells, myeloid‑derived suppressor cells and M2‑polarized tumor‑associated macrophages restrain effector responses through cytokines such as IL‑10 and TGF‑β, through checkpoint signaling and through metabolic interference [82, 88, 89, 90, 91]. Tumor‑associated macrophages also promote angiogenesis and tissue remodeling via VEGF, CXCL8, and matrix metalloproteinases [80, 92]. In contrast, tertiary lymphoid structures containing B cells and T cells correlate with local adaptive immunity and more inflamed tumor states [77]. The equilibrium between effector and suppressive populations therefore profoundly influences whether the microenvironment remains cold or shifts toward immune activation.

### 3.4 Physical, Vascular, and Metabolic Barriers That Maintain Immune Exclusion

Beyond cellular composition, the cold tumor microenvironment is shaped by structural and metabolic barriers that restrict immune infiltration and effector function [93, 94]. Cancer‑associated fibroblasts deposit extracellular matrix components including collagen and hyaluronic acid, generating dense stromal architecture that confines lymphocytes outside tumor nests [93, 95]. These fibroblasts also secrete factors such as CXCL12 and TGF‑β, which further promote immune exclusion and dysfunction [96, 97, 98]. Extracellular matrix remodeling, mediated in part by matrix metalloproteinases and DDR1 signaling, reinforces this physical barrier [21, 99].

Vascular abnormalities impose additional constraints. Aberrant VEGF and TGF‑β signaling impair expression of endothelial adhesion molecules such as ICAM and VCAM, thereby limiting lymphocyte trafficking across the vascular wall [94]. Vascular dysfunction also induces hypoxia, which stabilizes hypoxia‑inducible factors (HIFs), upregulates PD‑L1 expression, suppresses antigen presentation, and promotes recruitment of suppressive myeloid cells and regulatory T cells [100, 101, 102]. Metabolic stress adds another layer of constraint. Lactate accumulation acidifies the microenvironment and impairs T‐cell and NK‐cell cytotoxicity, whereas nutrient competition and MDSC‑mediated arginase and iNOS activity deprive lymphocytes of metabolites required for effector function [90, 103, 104]. Stromal remodeling, vascular dysfunction, hypoxia, and metabolic competition thus converge to establish the physical and biochemical foundation for sustained immune exclusion.

### 3.5 Signaling Networks That Stabilize the Cold Tumor Ecosystem

These ecological features are orchestrated by multiple signaling networks that integrate stromal, immune and metabolic cues. TGF‑β signaling suppresses T‐cell and NK‐cell activity, promotes accumulation of regulatory T cells and M2 macrophages, and drives epithelial–mesenchymal transition with concomitant loss of immunogenicity. NF‑κB signaling promotes recruitment of suppressive myeloid populations and upregulates PD‑L1 expression [101, 105, 106, 107]. Suppressed cGAS–STING signaling reduces Type I interferon production and attenuates immune recruitment. IL‑10 and STAT3 signaling further impairs antigen‑presenting cell maturation and T‐cell function while expanding suppressive populations [108, 109, 110, 111, 112]. These pathways do not operate in isolation; they form an interconnected regulatory network that reinforces the cold tumor state and constrains therapeutic.

**FIGURE 2:**
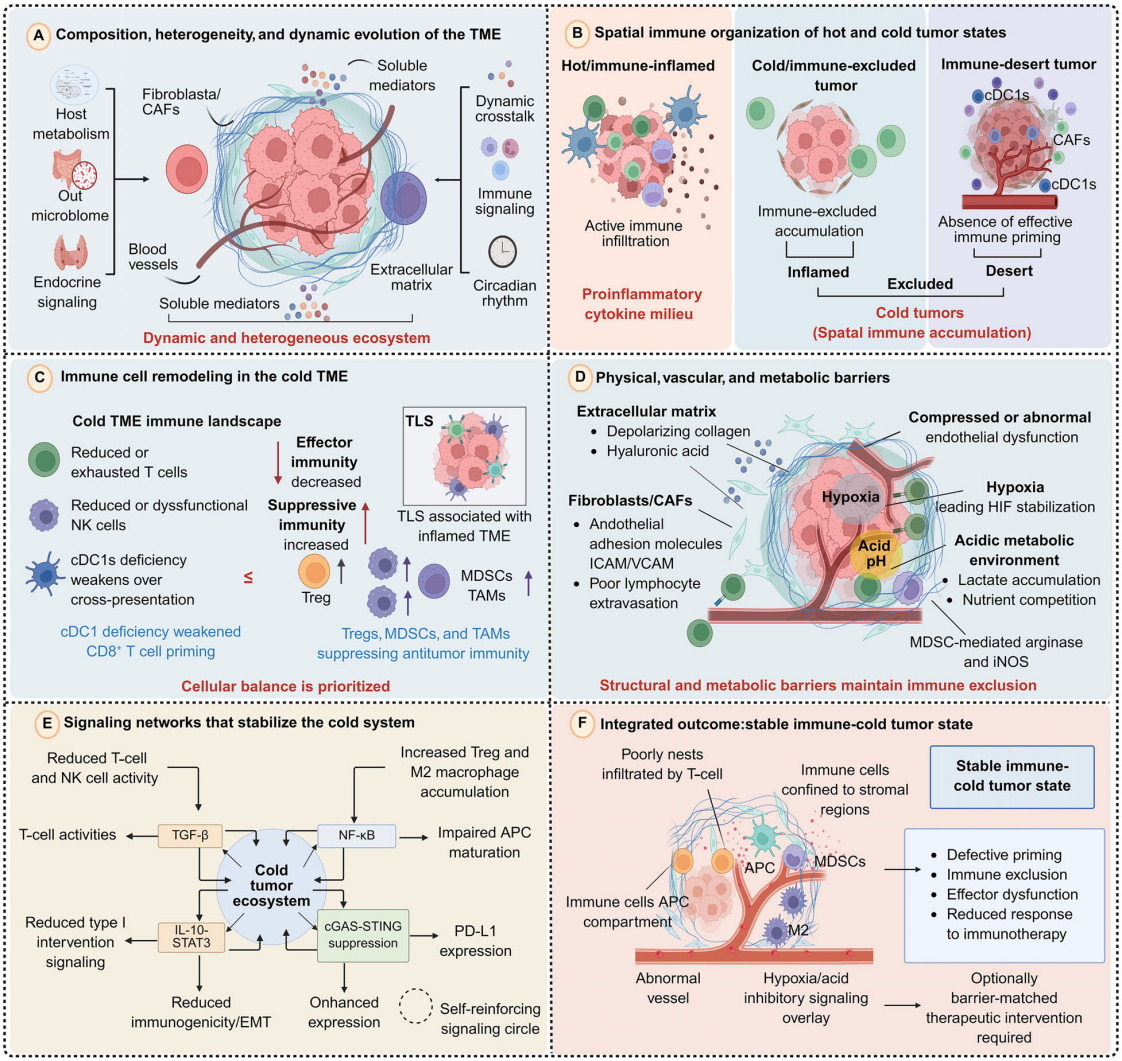
The tumor microenvironment as an ecosystem stabilizing immune‐cold tumor states. The TME integrates stromal, vascular, immune, metabolic, and systemic cues that shape spatial immune organization and sustain immune‐cold tumor states. The numbered panels indicate: (A) The dynamic and heterogeneous composition of the TME, including CAFs, blood vessels, ECM, soluble mediators, and systemic influences; (B) Spatial immune patterns ranging from hot/immune‐inflamed tumors to cold/immune‐excluded and immune‐desert states; (C) Immune‐cell remodeling in the cold TME, characterized by reduced effector immunity and increased suppressive immune populations; (D) Physical, vascular, and metabolic barriers that restrict lymphocyte trafficking and maintain immune exclusion; (E) Signaling networks, including TGF‐β, NF‐κB, IL‐10–STAT3, and suppressed cGAS–STING signaling, that reinforce the cold tumor ecosystem; (F) The integrated outcome of a stable immune‐cold tumor state with defective priming, immune exclusion, and effector dysfunction. APC, antigen‐presenting cell; CAF, cancer‐associated fibroblast; cDC1, type 1 conventional dendritic cell; ECM, extracellular matrix; MDSC, myeloid‐derived suppressor cell; NK, natural killer; TAM, tumor‐associated macrophage; TLS, tertiary lymphoid structure; TME, tumor microenvironment.

Collectively, the tumor microenvironment integrates cellular, structural, metabolic, and signaling barriers into a self‑reinforcing ecosystem (Figure 2). This ecosystem does more than accompany immune evasion; it underpins its persistence and amplification. Characterizing this organization is essential for devising mechanism‑guided and biomarker‑informed strategies to convert cold tumors into hot tumors.

## 4 Strategies to Transform Cold Tumors Into Hot Tumors

**FIGURE 3:**
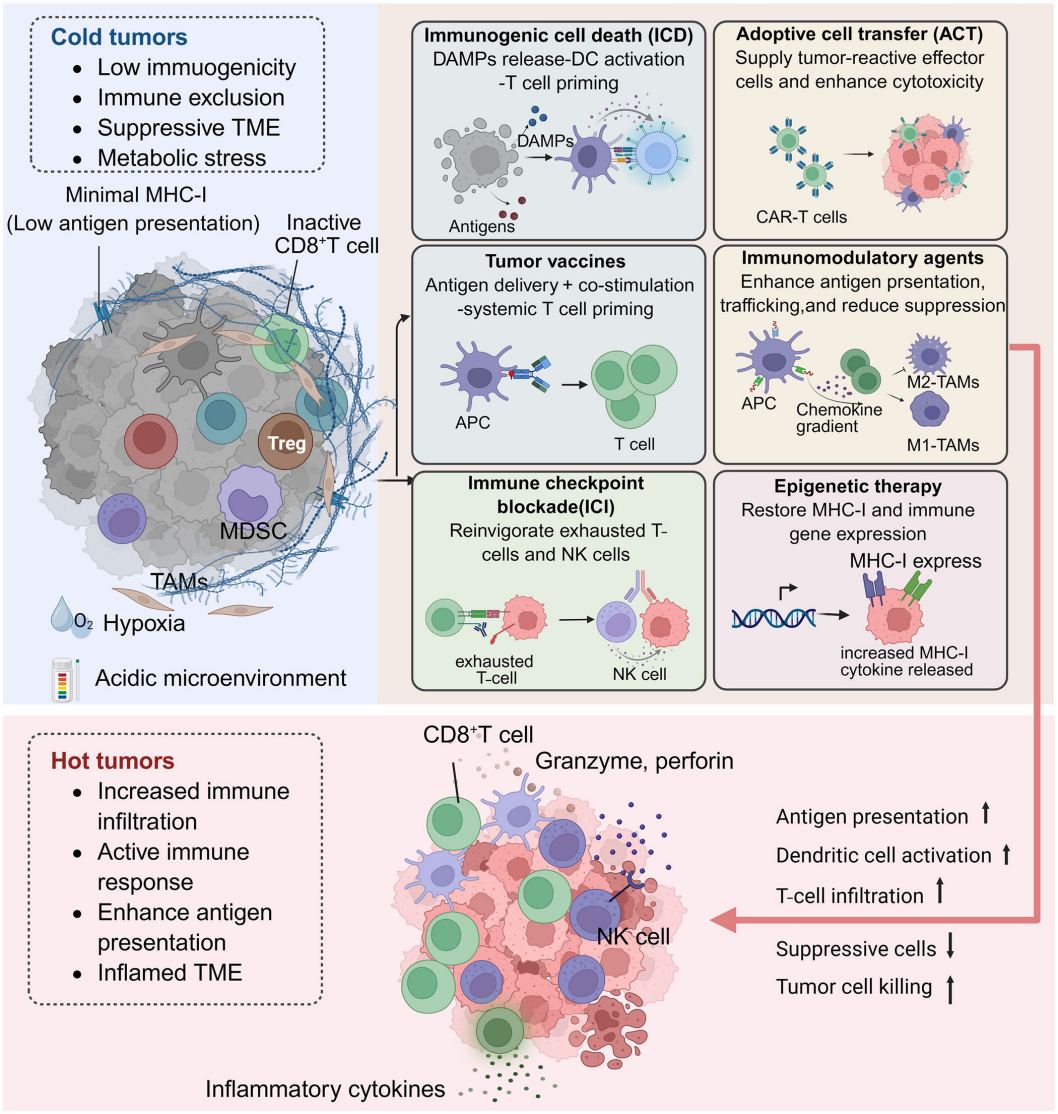
Integrated therapeutic strategies for converting cold tumors into hot tumors. Cold tumors are characterized by low immunogenicity, limited antigen presentation, immune exclusion, suppressive immune‐cell infiltration, hypoxia, and metabolic stress. The figure summarizes major therapeutic approaches used to promote cold‐to‐hot tumor conversion, including immunogenic cell death‐inducing therapies, tumor vaccines, immune checkpoint blockade, adoptive cell transfer, immunomodulatory agents, and epigenetic therapy. These interventions enhance antigen presentation, dendritic‐cell activation, immune‐cell infiltration, and effector‐cell cytotoxicity, while reducing suppressive immune programs. The integrated outcome is an inflamed hot tumor state with increased CD8^+^ T‐cell and NK‐cell activity, inflammatory cytokine production, and improved tumor‐cell killing. ACT, adoptive cell transfer; APC, antigen‐presenting cell; CAR‐T, chimeric antigen receptor T cell; DAMP, damage‐associated molecular pattern; DC, dendritic cell; ICD, immunogenic cell death; ICI, immune checkpoint inhibitor; MDSC, myeloid‐derived suppressor cell; MHC‐I, major histocompatibility complex Class I; NK, natural killer; TAM, tumor‐associated macrophage; TME, tumor microenvironment; Treg, regulatory T cell.

Cold tumors are sustained by multiple interdependent barriers: impaired immune sensing and priming, restricted immune cell access, metabolic hostility, and effector dysfunction within the microenvironment. These barriers often coexist and interact, so organizing therapeutic strategies solely by modality does not adequately capture the biology of cold‑to‑hot conversion. We therefore adopt a barrier‑oriented framework, discussing interventions according to the dominant rate‑limiting barriers they are designed to overcome. Major modalities relevant to this conversion, including ICD‑inducing agents, immune checkpoint inhibitors, tumor vaccines, ACT, oncolytic viruses, immunomodulatory agents, epigenetic modifiers, platinum‑based chemotherapy, and rational combinations, are evaluated against the specific barriers they target. Successful conversion demands restoration of tumor immunogenicity, facilitation of immune cell infiltration, preservation of immune fitness within hostile niches, and reprogramming of the microenvironment to support durable antitumor responses (Figure 3).

### 4.1 Restoring Tumor Visibility and Immune Priming

In cold tumors, particularly immune‑desert lesions, the primary barrier is failed immune recognition. Strategies must therefore restore tumor visibility and re‑establish the initial steps of the cancer–immunity cycle.

#### 4.1.1 Epigenetic Reprogramming to Restore Immune Visibility

Epigenetic therapies directly counter transcriptional silencing that impairs antigen presentation and immune priming. DNA methyltransferase inhibitors and histone deacetylase inhibitors can restore MHC Class I expression and antigen‑processing machinery components, including B2M and TAP2, by reversing repressive chromatin marks [7, 113, 114, 115, 116, 117, 118, 119]. EZH2 inhibition relieves PRC2‑mediated suppression of antigen presentation programs, whereas targeting chromatin regulators such as SETDB1 and KDM5D can enhance CD8^+^ T‐cell‑mediated tumor recognition [120, 121, 122, 123]. These agents reprogram the chromatin landscape to support antigen display and immune recognition, thereby reinstating tumor immunogenicity.

#### 4.1.2 Immunogenic Cell Death as a Mechanism for Immune Priming

ICD provides a route to restore immune priming in poorly inflamed tumors. When tumor‐cell death is accompanied by release of damage‑associated molecular patterns (DAMPs), including calreticulin, HMGB1, and ATP, dying cells supply both antigenic material and endogenous adjuvant signals that promote dendritic‐cell maturation and T‐cell activation [124, 125]. ICD can arise from multiple regulated cell death pathways, including apoptosis, necroptosis, pyroptosis, and ferroptosis, each of which may contribute to immune activation under appropriate biological and therapeutic conditions.

Several anticancer agents can induce or enhance ICD. Selected platinum‑based compounds, particularly oxaliplatin, promote DAMP release and augment immune activation, whereas cisplatin may enhance antigen release and cross‑presentation in specific contexts [125, 126]. In this setting, platinum‑based therapy is best viewed as one component of a broader immune‑conditioning strategy that links cytoreduction to improved tumor immunogenicity and increased susceptibility to subsequent immunotherapy.

#### 4.1.3 Tumor Vaccines to Rebuild Immune Priming

Tumor vaccines directly counter defective antigen presentation and priming in cold tumors. By delivering tumor‑associated antigens, neoantigens or shared tumor‑specific antigens together with appropriate adjuvants, vaccines can restore priming in tumors where spontaneous immune initiation is insufficient or absent [127]. These approaches hold particular promise for tumors with low baseline immunogenicity or substantial antigenic heterogeneity, as they can broaden immune recognition and promote systemic immune surveillance.

#### 4.1.4 Oncolytic Viruses as Platforms for In Situ Immune Priming

Oncolytic viruses induce tumor‐cell death while releasing antigens and danger signals, thereby promoting dendritic‐cell maturation and T‐cell activation [128, 129, 130, 131]. These agents bridge multiple barriers, including immune priming, immune cell recruitment, and local immune reprogramming, which makes them particularly attractive for tumors with limited spontaneous inflammation. Beyond in situ priming, they can also facilitate immune cell recruitment and microenvironmental remodeling, supporting their incorporation into rational combination strategies.

### 4.2 Restoring Immune Access and Effector Delivery

A second major obstacle in cold tumors is that immune cells often fail to infiltrate the tumor core, even after successful priming. Therapeutic strategies must therefore overcome physical exclusion and enable immune cell access to tumor nests.

#### 4.2.1 Vascular Normalization

Antiangiogenic agents can normalize tumor vasculature, improving endothelial adhesion, and blood flow to facilitate immune cell infiltration. The benefit of vascular normalization depends critically on timing. Excessive pruning may exacerbate hypoxia, whereas appropriately timed intervention creates a transient window that enhances immune cell trafficking and drug delivery [132, 133].

#### 4.2.2 Stromal Remodeling

Stromal remodeling strategies target pathways such as TGF‑β, DDR1, and CXCL12–CXCR4 to alleviate physical barriers that trap immune cells in the stromal compartment. These interventions facilitate immune cell penetration into the tumor core without inducing excessive fibrosis or tissue disorganization [21, 93, 94, 97, 98, 99, 134, 135, 136, 137]. The goal is selective remodeling of the tumor stroma to permit immune access and support therapeutic efficacy.

#### 4.2.3 Adoptive Cell Transfer for Effector Delivery Under Trafficking Constraints

ACT delivers tumor‑reactive immune cells directly, bypassing certain priming defects. In solid tumors, however, its efficacy is constrained less by the quality of the infused cells than by their access to, and persistence within, the malignant lesion. CAR‑T cells, TCR‑engineered cells and adoptively transferred NK cells must traverse the vascular wall, penetrate stromal barriers and survive within metabolically hostile niches. ACT should therefore be viewed as one component of a broader strategy that addresses both immune access and effector fitness [138, 139].

### 4.3 Overcoming Metabolic Constraints on Immune Fitness

TME comprises diverse cell types and is both metabolically and spatially heterogeneous. Metabolic stress varies across TME regions, driven largely by spatial heterogeneity: Cellular populations compete for limited oxygen and nutrients in areas with sparse vasculature or high metabolic demand. This competition leads to accumulation of metabolic waste and nutrient depletion. Nutrient availability within the TME is further influenced by systemic and tissue‑level factors, including tumor type, the site of the primary lesion, host diet and nutritional status. Metabolic plasticity enables cells within the TME to utilize alternative nutrients when preferred substrates become scarce. Such heterogeneity not only fuels proliferation of transformed cells, but also facilitates metastatic progression by shaping multiple steps of the metastatic cascade, both within tumor cells and in the local TME during dissemination. Even when immune cells successfully infiltrate the tumor, their function remains constrained by this metabolically hostile and heterogeneous landscape. Sustaining immune fitness within a spatially and metabolically challenging TME therefore represents a critical therapeutic imperative [140].

#### 4.3.1 Metabolic Reprogramming to Preserve Effector Function

Metabolic interventions, including HIF inhibitors, lactate transport inhibitors, glutaminase inhibitors and adenosine pathway modulators, target hypoxia, acidosis and nutrient competition within the tumor microenvironment [141, 142, 143]. These agents aim to restore a metabolic environment that supports T‐cell and NK‐cell function.

#### 4.3.2 Clinical and Pharmacologic Constraints

Metabolic interventions can target cancer‐cell‐intrinsic dependencies, tumor‐microenvironmental constraints or systemic host metabolism. Current development focuses on nucleotide biosynthesis, bioenergetic pathways and redox control. Nucleotide‐synthesis inhibitors are the most clinically advanced, whereas agents targeting mutant IDH, GPX4, or NAMPT remain supported mainly by early clinical or preclinical evidence. Metabolic targeting may also restore sensitivity to chemotherapy, radiotherapy and immunotherapy by altering nutrient availability, cytokine gradients, redox balance or lipid‐mediated drug uptake [144, 145].

Clinical translation remains inconsistent. Many metabolic pathways are shared by malignant cells, stromal compartments and normal proliferative tissues, resulting in narrow therapeutic windows and dose‐limiting toxicity. Nonselective inhibition may also impair effector T cells, NK cells, and antigen‐presenting cells. In addition, metabolic dependencies vary across tumor types, patients and intratumoral regions, whereas metabolic plasticity enables compensatory pathway use [144, 145]. Future strategies will require biomarker‐driven patient selection, pharmacodynamic monitoring of target engagement and locoregional delivery to achieve localized, durable and immune‐supportive metabolic modulation.

### 4.4 Reprogramming the Tumor Microenvironment

In some cold tumors, immune cells are present but rendered dysfunctional by chronic inhibitory signaling and suppressive microenvironmental circuits. Strategies must therefore restore effector function and remodel these regulatory networks within the tumor microenvironment [8].

#### 4.4.1 Checkpoint Blockade

Immune checkpoint blockade therapies, including anti‐PD‐1, anti‐PD‐L1, and anti‐CTLA‐4 antibodies, restore T‐cell signaling and effector function in exhausted lymphocytes, thereby enabling more sustained immune responses [55, 146, 147, 148]. Additional checkpoint inhibitors targeting LAG‐3, TIGIT, and TIM‐3 may further restore T‐cell function by blocking complementary inhibitory pathways [149, 150, 151, 152], as shown in Figure 1.

#### 4.4.2 Immunomodulatory Agents

Immunomodulatory agents, such as cGAS–STING agonists and Toll‐like receptor ligands, enhance dendritic‐cell function, Type I interferon signaling, and local inflammatory responses [153]. These agents promote antigen presentation and T‐cell recruitment, thereby strengthening immune activation within the TME, as shown in Figure 2.

### 4.5 Rational Combination Strategies

Because cold tumors are maintained by multiple interacting bottlenecks, combination therapy is often required. The goal is not simply to increase treatment intensity, but to coordinate interventions across distinct rate‐limiting barriers [154, 155].

#### 4.5.1 Barrier‐Matched Combinations

Combinations should be aligned with the dominant barrier architecture of each tumor. In tumors where impaired immune sensing and priming predominate, epigenetic reprogramming and ICD‑inducing agents, including selected platinum‑based regimens in appropriate settings, may be paired with checkpoint blockade. In immune‑excluded tumors, vascular normalization and stromal remodeling may be needed before or alongside T‑cell‑directed therapies to improve intratumoral access. In tumors dominated by suppressive myeloid or regulatory programs, microenvironment‑reprogramming strategies should be integrated with interventions that restore immune recognition, trafficking and effector function [156].

#### 4.5.2 Adapting to Tumor Heterogeneity

Combination strategies must also contend with spatial and temporal heterogeneity. Spatial heterogeneity can generate coexisting hot and cold regions within the same lesion, supporting combinations that link local remodeling with systemic immune activation [60, 66, 67, 74, 76, 77, 90]. Temporal heterogeneity can drive adaptive resistance, underscoring the need for dynamic and adjustable regimens.

#### 4.5.3 Biomarker‐Guided Combination Strategies

Successful cold‐to‐hot conversion will depend on biomarker‐guided, sequentially adaptable combinations tailored to the dominant barrier architecture of each tumor. The objective is to dismantle the specific ecological structures that sustain immune‐cold disease, rather than relying on a single, universally effective regimen [157, 158].

## 5 Preclinical Models and Evidence for Converting Cold Tumors

Preclinical studies have identified the biological constraints that sustain cold tumors and have tested whether these constraints can be overcome. Several principles have emerged: defective immune priming can be reinstated [159]; immune exclusion can be partially relieved through stromal and vascular remodeling [160]; immune cell fitness can be improved within metabolically hostile niches [161]; and durable conversion generally requires coordinated modulation of multiple rate‑limiting steps in the cancer–immunity cycle [8, 162]. The most informative studies demonstrate concurrent restoration of antigen presentation, effector‐cell access, effector competence and sustained tumor control, rather than isolated increases in inflammatory signaling or CD8^+^ T‐cell density.

The translational relevance of these findings is heavily influenced by model design. Many commonly used systems are transplantable, antigen‑enriched, rapidly progressive, and experimentally synchronized [163]. These models are useful for detecting immune activation and short‑term inflammatory responses, but they incompletely recapitulate the stromal burden, spatial restriction and evolutionary persistence of human cold tumors. Human tumors differ from these models in several respects: they develop over long periods under fluctuating immune pressure and therapeutic exposure, acquire greater genomic and phenotypic heterogeneity, and form denser and more spatially organized stromal architectures than most murine transplant models [164]. Syngeneic models are myeloid‑biased, whereas human tumors show greater T‑cell variability. TME state, not tissue origin, should guide model selection. RENCA is an exception. The conserved T_3_–My_2_ axis correlates with survival [164].

Preclinical systems may therefore overstate the feasibility of cold‑to‑hot conversion and may underestimate the extent to which stromal complexity, interlesional heterogeneity, and immunoediting constrain therapeutic efficacy in patients.

### 5.1 Preclinical Restoration of Tumor Visibility and Immune Priming

Failed immune initiation is among the most consistently observed features of cold tumors in preclinical systems. This upstream defect offers a logical entry point for evaluating therapeutic conversion, because downstream interventions are unlikely to achieve durable benefit without restoring antigen sensing, dendritic‐cell activation, and T‐cell priming. Preclinical efforts have focused on direct innate pathway activation, vaccine‑based restoration of tumor‑specific priming, in situ inflammatory priming, and selected epigenetic strategies that restore tumor visibility while promoting permissive inflammatory cues. Representative preclinical and translational strategies for cold‑to‑hot tumor conversion, grouped by their targeted immune barriers, are summarized in Table 1.

**TABLE 1:** Core strategies for converting cold tumors to hot tumors.

Dominant barrier	Conversion strategy	Context/study design	Evidence/cold‐to‐hot implication	Refs.
**Antigenicity/priming deficit—Increase antigen visibility, antigen release, or tumor‐specific T‐cell priming**.				
Epigenetic silencing of antigen presentation	EZH2 inhibition with T‐cell‐engaging immunotherapy	Melanoma; preclinical test of EZH2 adaptive resistance.	EZH2 blockade restored antigen presentation and immune visibility, and synergized with anti‐CTLA‐4/IL‐2; preclinical evidence for epigenetic resensitization.	[40]
Low neoantigen‐driven priming	Autogene cevumeran + atezolizumab + mFOLFIRINOX	Resected PDAC; NCT04161755; Phase 1; safety, RFS and OS.	Vaccine‐induced T cells correlated with prolonged RFS and persistent CD8+ clones; small cohort but demonstrates de novo priming in a cold tumor type.	[165]
Limited priming in low‐mutational‐burden RCC	Personalized neoantigen vaccine ± local ipilimumab	High‐risk resected clear‐cell RCC; NCT02950766; Phase I; safety, T‐cell response and recurrence‐free status.	All 9 patients generated T‐cell responses and had no recurrence at reported follow‐up; very small adjuvant cohort requiring validation.	[166]
Suboptimal CD8+ T‐cell priming	Vaccine/PD‐1 sequencing; PD‐1+CD38hi biomarker	Anti‐PD‐1‐resistant models and patient samples; mechanistic/translational.	PD‐1 blockade under suboptimal priming induced dysfunctional PD‐1 + CD38hi CD8+ cells, whereas optimal priming reversed resistance; supports sequencing of priming and checkpoint release.	[167]
Epigenetically restricted immune activation	Decitabine followed by immune checkpoint inhibition	PDAC mouse models; preclinical TME‐ remodeling study.	DAC increased T‐cell infiltration and altered myeloid populations; DAC plus ICI further increased immune infiltration; supports epigenetic sensitization, but human validation is lacking.	[168]
Insufficient patient‐specific neoantigen priming	mRNA‐4157/V940 + pembrolizumab	Completely resected high‐risk melanoma; NCT03897881; randomized Phase 2b; RFS and distant metastasis‐free survival.	18‐month RFS 79% versus 62%; recurrence/death 22% versus 40%; supports de novo neoantigen immunity but needs Phase III confirmation.	[169]
Weak tumor‐specific immunity	GNOS‐PV02 + plasmid IL‐12 + pembrolizumab	Advanced HCC after multikinase inhibitor therapy; NCT04251117; Phase 1/2; safety, immunogenicity and efficacy.	ORR 30.6%; neoantigen‐specific T‐cell responses in 19/22 evaluable patients; single‐arm study suggesting vaccine‐enhanced PD‐1 sensitization.	[170]
Need for antigen release and immune conditioning	Pembrolizumab + pemetrexed‐platinum chemotherapy	First‐line metastatic nonsquamous NSCLC without EGFR/ALK alterations; NCT02578680; Phase 3; OS and PFS.	OS HR 0.60 and PFS HR 0.50; 5‐year OS 19.4% versus 11.3%; supports chemo‐immunotherapy, although direct immune conversion was not proven.	[171]
Chemotherapy‐associated immune conditioning	Atezolizumab + platinum‐pemetrexed chemotherapy	Stage IV nonsquamous NSCLC without EGFR/ALK alterations; NCT02657434; Phase 3; OS and investigator‐assessed PFS.	PFS 7.6 versus 5.2 months; OS numerically improved but not statistically significant; immune‐conditioning rationale remains indirect.	[172]
Radiotherapy‐associated immune conditioning	Durvalumab after sequential chemoradiotherapy	Stage III unresectable NSCLC after platinum‐based sCRT; NCT03693300; Phase 2; safety, PFS and OS.	Grade 3/4 possibly related AEs within 6 months: 4.3%; median PFS 10.9 months; 12‐month OS 84.1%; single‐arm evidence for consolidating CRT‐conditioned tumors.	[173]
**Defective innate sensing/local inflammatory initiation—Initiate innate inflammation and convert the tumor into an in situ priming site**.				
Defective innate sensing/Type I IFN induction	MK‐1454 STING agonist with anti‐PD‐1 rationale	Syngeneic tumor models; preclinical drug‐discovery study.	Intratumoral MK‐1454 induced cytokine upregulation and antitumor activity, enhanced with anti‐PD‐1; clinical efficacy not reported.	[174]
Insufficient innate amplification after radiotherapy	TLR7/8 agonist R848 + SBRT	Murine PDAC models; preclinical test of SBRT‐induced immunity.	R848 altered the immune TME and enhanced SBRT antitumor efficacy; systemic translation and toxicity remain unresolved.	[175]
Inefficient DC recruitment and cross‐priming	In situ vaccination: Flt3L + local radiotherapy + poly‐ICLC	Indolent non‐Hodgkin lymphoma; NCT01976585; Phase I; recruit, load and activate intratumoral DCs.	Induced DC expansion, T‐cell infiltration and regression of untreated lesions; small cohort and checkpoint upregulation may limit durability.	[176]
Poor local antigen release and immune activation	T‐VEC versus GM‐CSF	Unresected stage IIIB‐IV melanoma; NCT00769704; Phase III; DRR, OS, and ORR.	DRR 16.3% versus 2.1%; ORR 26.4% versus 5.7%; median OS 23.3 versus 18.9 months; lesion accessibility limits delivery.	[130]
Limited local immune activation	T‐VEC + pembrolizumab	Locally advanced/metastatic sarcoma; Phase 2; ORR at 24 weeks; NCT not reported.	Best ORR at 24 weeks 30%; overall ORR 35%; no grade 4 TRAEs or treatment‐related deaths; single‐institution, heterogeneous histologies.	[177]
Cold glioblastoma microenvironment	DNX‐2401 followed by pembrolizumab	Recurrent glioblastoma; NCT02798406; Phase 1/2; safety, ORR, 12‐month OS, and exploratory immune analyses.	ORR 10.4%; 12‐month OS 52.7%; clinical benefit 56.2%; response associated with immune infiltration/checkpoint expression; primary efficacy endpoint not met.	[178]
Poor inflammatory priming in DIPG	DNX‐2401 followed by radiotherapy	Newly diagnosed pediatric DIPG; dose‐escalation study; safety/adverse events, OS, response, and immune correlates.	Median OS 17.8 months; tumor‐size reduction in nine patients; PR in three and SD in eight; very small nonrandomized cohort.	[179]
Need to improve PD‐1 response by local priming	T‐VEC + pembrolizumab versus placebo + pembrolizumab	Stage IIIB‐IVM1c unresectable melanoma, anti‐PD‐1‐naive; NCT02263508; randomized Phase III; PFS and OS.	No significant PFS or OS improvement; ORR 48.6% versus 41.3%; local priming likely requires patient selection or barrier matching.	[180]
**Immune exclusion/restricted access—Remodel vascular, stromal, or chemokine barriers to enable immune‐cell entry**.				
CXCL12–CXCR4 stromal‐vascular crosstalk	CXCR4/CXCL12 targeting as antiangiogenic and TME‐ remodeling strategy	Prostate cancer; multiomic human profiling with xenograft validation; translational/preclinical.	Tumor endothelial cells showed vascular, collagen and ECM‐remodeling programs with high CXCL12 in arterial TECs; points to vascular/stromal barrier targeting.	[181]
Peritumoral adipose tissue as an immune‐cell sink	Target adipose‐tumor interaction or CXCL12–CXCR4 axis	Colorectal cancer; human single‐cell analysis and mouse validation; translational/preclinical.	tVAT recruited CXCR4+ immune cells via CXCL12; tVAT removal or CXCL12 blockade increased tumor infiltration and suppressed growth; clinical validation needed.	[182]
VEGF‐driven abnormal vasculature	Avelumab + axitinib	Previously untreated advanced RCC; NCT02493751; open‐label multicenter Phase Ib; DLT, safety, ORR, DOR, PFS and OS.	*n* = 55; ORR 60.0%; CR 10.9%; median DOR 35.9 months; median PFS 8.3 months; 5‐year OS 57.3%; single‐arm evidence for PD‐L1/VEGFR cotargeting.	[183]
CXCL12–CXCR4‐mediated T‐cell exclusion	BL‐8040/motixafortide + pembrolizumab, with or without chemotherapy	Metastatic PDAC; NCT02826486; Phase IIa; ORR, OS, DCR, safety and immunobiology.	Cohort 1: DCR 34.5%, mOS 3.3 months; cohort 2: ORR 32%, DCR 77%, median DOR 7.8 months; increased CD8+ effector infiltration, but randomized validation is needed.	[184]
VEGF‐mediated vascular dysfunction	TACE + ICIs + anti‐VEGF antibody/TKIs	Advanced HCC, first‐line real‐world treatment; NCT05332821; target‐trial emulation; OS, PFS, ORR and safety.	*n* = 1244; OS 22.6 versus 15.9 months; PFS 9.9 versus 7.4 months; ORR 41.2% versus 22.9%; retrospective emulation with residual confounding risk.	[185]
Vascular normalization versus hypoxia	Antiangiogenic agents combined with ICIs	Multiple tumor types; review of completed Phase III trials.	VEGF blockade may improve DC maturation, CD8+ infiltration and vascular normalization, but can increase hypoxia or reduce ICI ingress; benefit depends on context, dose and schedule.	[186]
**Suppressive niche/effector dysfunction or poor persistence—Reinvigorate, preserve or engineer effector cells after tumor entry**.				
LAG‐3/PD‐1‐mediated T‐cell exhaustion	Relatlimab + nivolumab versus nivolumab	Previously untreated metastatic/unresectable melanoma; NCT03470922; randomized Phase 2/3; blinded independent PFS.	Median PFS 10.1 versus 4.6 months; HR 0.75; grade 3/4 TRAEs 18.9% versus 9.7%; dual blockade reinvigorates T cells but adds toxicity.	[187]
Metabolic stress and immune‐cell exhaustion	Target metabolic checkpoints to enhance ICB and ACT	Broad cancer immunotherapy context; review of immune‐cell metabolic checkpoints.	Metabolic modulation may improve CD8+ T‐cell fitness, ICB efficacy and ACT persistence in preclinical settings; clinical translation is context‐dependent.	[188]
Poor ACT persistence and metabolic vulnerability	Metabolic engineering of TIL, CAR‐T, TCR‐T and NK‐cell therapies	ACT in solid tumors.	May improve memory‐like states, mitochondrial function and effector persistence; mostly preclinical and still constrained by trafficking, exhaustion and suppressive TME.	[188]
Mechanical constraints on T‐cell function	T‐cell engineering through mechanosensitive force‐bearing receptors	T‐cell engineering and ACT; review.	Mechanical cues regulate activation, cytokine production, metabolism and migration; clinical application remains early and endpoints are not standardized.	[189]
Suppressed immunity in the native tumor context	Neoadjuvant nivolumab with or without relatlimab	Resectable NSCLC; NCT04205552; randomized Phase 2; surgery feasibility, pathological/radiographic response, and survival.	Surgery feasibility met in all patients; MPR 27% with nivolumab and 30% with nivolumab‐relatlimab; grade ≥ 3 AEs 10% and 13%; follow‐up immature.	[190]
Timing and personalization of perioperative ICI	Neoadjuvant/perioperative ICI strategies	Early‐stage melanoma, RCC, and NSCLC.	Neoadjuvant/perioperative ICI can improve pathological response and event‐free outcomes in selected settings; optimal sequencing and adjuvant contribution remain unsettled.	[191]
PD‐1/PD‐L1 resistance and immune evolution	PD‐1/PD‐L1 resistance mechanisms and sensitizing strategies	Multiple tumor types.	Resistance involves abnormal PD‐1/PD‐L1 expression, immune‐related pathways, suppressive TME and T‐cell dysfunction; explains why checkpoint release alone is insufficient.	[192]

Abbreviations: ACT, adoptive cell therapy; AEs, adverse events; CR, complete response; DAC, decitabine; DC, dendritic cell; DLT, dose‐limiting toxicity; DOR, duration of response; DRR, durable response rate; GBM, glioblastoma; HCC, hepatocellular carcinoma; ICB, immune‐checkpoint blockade; ICI, immune‐checkpoint inhibitor; MPR, major pathological response; ORR, objective response rate; OS, overall survival; PDAC, pancreatic ductal adenocarcinoma; PFS, progression‐free survival; RCC, renal cell carcinoma; RFS, recurrence‐free survival; RT, radiotherapy; SBRT, stereotactic body radiotherapy; TACE, transarterial chemoembolization; TME, tumor microenvironment; T‐VEC, talimogene laherparepvec.

#### 5.1.1 Innate Agonism and Partial Recovery of Priming

Direct activation of innate immune‑sensing pathways provides an immediate test of whether cold tumors retain the capacity to re‑enter the cancer–immunity cycle. These approaches can restore dendritic‐cell activation and T‐cell recruitment, while also revealing compensatory suppressive programs that emerge after inflammation is re‑established. Agonists of the cGAS–STING pathway, including cGAMP and MK‑1454, induce Type I interferon production, promote dendritic‐cell maturation and enhance CD8^+^ T‐cell recruitment in models such as MC38 and B16F10 melanoma [174]. These effects are often accompanied by compensatory inhibitory responses. In ovarian cancer models, STING activation upregulates PD‑L1 expression [193], whereas in HPV‑associated tongue squamous cell carcinoma, it may increase Treg infiltration unless combined with checkpoint blockade [194]. Innate agonism can therefore reinitiate antitumor immunity, but rarely achieves durable conversion as a single agent.

Delivery strategy further shapes activity. pH‑responsive PLGA nanoparticles loaded with the TLR7/8 agonist R848 activate macrophages and dendritic cells in melanoma [195], and nanocarrier systems that improve intratumoral retention enhance the activity of STING‑ or dsRNA‑based agonists in breast cancer, melanoma, and pancreatic cancer models [196, 197]. Efficacy therefore depends not only on pathway selection, but also on whether immune stimulation can be spatially restricted to the tumor or its draining lymphoid compartment.

#### 5.1.2 Vaccination and Restoration of Tumor‐Specific Priming

Vaccination addresses the same upstream defect through a more antigen‑directed mechanism. Its primary value lies in rebuilding tumor‑specific recognition rather than broadly imposing inflammatory activation. Neoantigen‑based and peptide‑based vaccines expand tumor‑specific T‑cell repertoires in preclinical models of renal cell carcinoma, pancreatic cancer, melanoma and glioblastoma, and in some settings reduce Treg abundance or limit the emergence of dysfunctional PD‑1^+^CD38ʰ^i^ CD8^+^ T‑cell states [167, 198, 199, 200, 201]. Efficacy is shaped by the adjuvant context, with TLR‑ and STING‑based adjuvants strengthening dendritic‐cell activation, chemokine induction and Th1 polarization [175, 202, 203, 204]. Vaccines can restore tumor‑specific priming, but as single agents they do not resolve downstream barriers to trafficking, immune exclusion or effector dysfunction.

#### 5.1.3 In Situ Inflammatory Priming

In situ priming converts the tumor itself into a site of antigen release and immune activation. This strategy is particularly relevant when systemic priming is insufficient but local inflammatory reprogramming remains achievable. Oncolytic viruses such as T‑VEC induce ICD and DAMP release in melanoma models [130, 205]. Local chemotherapy combined with innate agonists, Flt3L‑based intratumoral priming, intratumoral BCG and thermal ablation have shown similar capacity to induce broader antitumor immunity in colorectal, pancreatic, lung, and breast cancer models [176, 206, 207, 208]. Microbiota‑directed interventions operate along the same principle [209, 210]. Nevertheless, induction of local inflammation is easier to demonstrate experimentally than durable control of tumors with structural exclusion, metabolic hostility, and evolutionary heterogeneity.

#### 5.1.4 Epigenetic Restoration of Tumor Visibility With Permissive Inflammatory Rewiring

Epigenetic therapy contributes to microenvironmental remodeling, but its preclinical relevance is closely tied to restoring tumor visibility and antigen‑presenting capacity. In ovarian cancer, combined DNMT and EZH2 inhibition increases CXCL9 and CXCL10 expression and enhances CD8^+^ T‑cell infiltration [211]; in melanoma, EZH2 inhibition reverses epigenetic silencing of CXCL9 [40]. In colorectal cancer, 5‑azacytidine induces viral mimicry through the MDA5–MAVS–IRF7 pathway, increasing interferon production and immune infiltration [212]. In small‑cell lung cancer, EZH2 inhibition restores MHC‑I expression [213, 214], linking epigenetic reprogramming to both chemokine restoration and recovery of tumor visibility. Additional studies position epigenetic therapy at the intersection of antigen restoration, chemokine induction, and myeloid reprogramming [215, 216].

### 5.2 Preclinical Restoration of Immune Access and Delivery Constraints

Restoration of immune priming is insufficient when immune cells remain spatially restricted outside tumor nests. Preclinical studies in this area therefore test whether tissue architecture and vascular access can be reprogrammed to permit effective immune cell infiltration. Stromal and vascular remodeling addresses whether immune exclusion is structurally reversible, not merely immunologically inducible. Its value lies not simply in increasing lymphocyte entry, but in identifying barriers that can be selectively relaxed without destabilizing tissue organization.

In pancreatic ductal adenocarcinoma models, depletion of α‑SMA^+^ cancer‑associated fibroblasts improves vascular perfusion and increases CD8^+^ T‐cell infiltration, although excessive depletion may also expand Treg abundance [217]. In triple‑negative breast cancer and metastatic urothelial carcinoma, targeting DDR1 or TGF‑β disrupts collagen‑dependent exclusion and facilitates immune cell penetration [134, 135]. Lysyl oxidase inhibition reduces stromal stiffness and improves T‐cell motility in breast cancer [218]. Antiangiogenic normalization similarly reduces hypoxia and facilitates T‐cell entry in RCC and HCC models [219, 220]. These findings indicate that immune exclusion arises not only from defective priming, but also from abnormal tissue architecture and impaired vascular access. Representative preclinical and translational strategies for cold‑to‑hot tumor conversion, grouped by their targeted immune barriers, are summarized in Table 1.

### 5.3 Preclinical Preservation of Immune Fitness in Hostile Intratumoral Niches

Even when priming is restored and exclusion partially relieved, durable conversion depends on whether effector lymphocytes retain cytotoxicity, persistence, and resistance to exhaustion. This question matters because many preclinical interventions generate transient infiltration without durable functional immunity [221]. Evidence derives from interventions that directly modulate intratumoral metabolic stress and from studies that reinforce T‑cell‑intrinsic effector competence [222, 223].

#### 5.3.1 Metabolic Remodeling and Compartment‐Specific Effects

Metabolic remodeling adds further complexity, as the same pathway may exert opposing effects in tumor and immune compartments. Inhibition of LDHA or MCT1 reduces lactate accumulation and restores CD8^+^ T‐cell function in glycolysis‑dependent tumors [224, 225, 226]. Induction of ferroptosis through GPX4 inhibition or cystine deprivation can promote ICD and dendritic‐cell activation under appropriate conditions [227]. These effects are highly compartment‑dependent. In lipid‑rich melanoma models, protecting CD8^+^ T cells from ferroptosis via CD36 deletion preserves antitumor activity [228]. Effective remodeling of cold tumors therefore requires attention not only to immune cell recruitment, but also to local metabolic fitness and to the divergent consequences of targeting the same pathway in tumor and immune compartments.

#### 5.3.2 Intrinsic Reinforcement of Effector Competence

Manipulation of T‑cell‑intrinsic regulatory programs provides a direct way to assess whether dysfunctional effector states are reversible. These studies help define how exhaustion, persistence, and memory differentiation are regulated after immune cells enter the tumor. Prdm12 deficiency in CD8^+^ T cells, studied in the OT‑I/B16‑OVA system, enhances effector cytokine production, promotes memory‑precursor and effector‑like states, and limits exhaustion‑associated programs through the CGRP–RAMP1 axis and associated chromatin changes [229, 230, 231, 232, 233, 234]. Additional regulators, including HDAC1 and LSD1, have also been implicated in restoring cytotoxic gene expression and T‐cell persistence in TNBC and melanoma models [235, 236]. These findings support the concept that T‐cell competence can be strengthened through modulation of intrinsic regulatory programs. However, many of these observations derive from engineered antigen systems and therefore do not fully recapitulate the low antigenicity and clonal heterogeneity of naturally cold tumors.

### 5.4 Preclinical Reprogramming of Suppressive TME States

A separate body of preclinical work has addressed suppressive microenvironmental circuits that persist even after immune initiation has been restored. These studies examine whether suppressive macrophage states, checkpoint‑mediated restraint, stromal resistance, and related inhibitory programs can be sufficiently weakened to support productive antitumor immunity. The key question is not simply whether inflammation can be induced, but whether it can be sustained without being neutralized by compensatory suppressive responses [237].

In NSCLC and TNBC models, PD‑1 or PD‑L1 blockade combined with chemotherapy enhances MHC‑I expression, depletes suppressive populations and promotes ICD [238, 239]. In PDAC, addition of STING or NLRP3 agonists further augments immune activation and weakens stromal resistance [240]. Combinations of PD‑1 blockade with MET‑, EGFR‑, PARP‑, or VEGFR‑targeted therapies restore antigen presentation, improve dendritic‐cell maturation, or alleviate suppressive macrophage and metabolic states in context‑specific settings [241, 242, 243, 244, 245]. Vaccines directed against suppressive populations, including IDO‑ or Treg‑associated targets, extend immune reprogramming beyond tumor‑cell‑directed antigen recognition [246, 247, 248].

### 5.5 Preclinical Evidence for Barrier‐Matched Combinations

Combination strategies are required when correction of a single defect is insufficient. Their relevance lies in barrier complementarity rather than treatment intensity. Checkpoint blockade is more effective when paired with therapies that generate or broaden tumor‑reactive immunity. Dual‐checkpoint blockade, including PD‑1 plus LAG‑3, or TIGIT inhibition, enhances CD8^+^ T‐cell and NK‐cell function in melanoma and colorectal cancer models [187, 238, 249, 250, 251]. Local interventions can also be configured to amplify systemic immunity. In multiple models, radiotherapy combined with PD‑(L)1 blockade enhances immune trafficking to distant lesions and mitigates local exclusion [252, 253, 254], whereas neoantigen vaccines combined with checkpoint blockade improve priming while reducing Treg abundance [255].

Taken together, preclinical evidence supports the view that effective conversion of cold tumors requires coordinated intervention across multiple nonredundant barriers. The strongest studies link immune initiation with restoration of tissue access, preservation of effector competence, reprogramming of suppressive niches, and durable tumor control. These conclusions remain dependent on model design. Because many commonly used systems are transplantable, antigen‑enriched, and short‑duration models that do not fully reproduce stromal burden, lesion‑scale heterogeneity or prolonged immunoediting, current preclinical evidence may overestimate the feasibility of achieving durable cold‑to‑hot conversion in human tumors.

## 6 Clinical Trials and Case Studies for Converting Cold Tumors

Preclinical studies suggest that cold‑to‑hot tumor conversion can be pursued through restoration of antigen visibility and immune priming, activation of innate inflammatory signaling, relief of immune exclusion, preservation of effector‐cell fitness and reprogramming of suppressive microenvironmental states. Clinical translation, however, requires evidence that these barrier‑directed interventions generate durable patient benefit beyond transient immune activation, isolated biomarker changes or indirect signs of microenvironmental remodeling. Clinical benefit, therefore, should not be equated with bona fide cold‑to‑hot conversion.

As summarized in Table 1, current clinical evidence is uneven. The most robust support comes from established chemo‑immunotherapy and chemoradiotherapy regimens that provide immune conditioning in selected tumor contexts [119, 171, 172, 256, 257]. Antiangiogenic combinations offer comparatively mature evidence that vascular dysfunction, stromal restriction, and immune exclusion can be therapeutically modified [183, 220, 258]. By contrast, personalized vaccines, neoantigen‑based approaches, epigenetic regimens, oncolytic viruses, STING or TLR agonists, microbiota‑directed interventions, metabolic modulators and most cell‑based therapies remain supported mainly by early‑phase or signal‑generating studies, with limited direct evidence of durable conversion in prospectively defined cold tumor populations [21].

Available clinical studies are therefore best viewed as a graded map of translational maturity across barrier states, rather than uniform proof that cold tumors can be broadly converted. We next evaluate these strategies according to the dominant barriers they are designed to overcome, distinguishing established platforms from emerging approaches whose optimal sequencing, patient selection and long‑term clinical impact remain unresolved.

### 6.1 Clinical Restoration of Tumor Visibility and Immune Priming

Clinical studies targeting tumor immunogenicity examine whether therapies that increase antigen release, dendritic‐cell activation, or early inflammatory signaling can improve responsiveness in biologically cold tumors. Evidence is strongest for established immune‐conditioning backbones, whereas newer priming‐oriented strategies remain supported mainly by indirect or early‐phase clinical data.

#### 6.1.1 Established Immune‐Conditioning Backbones

Chemotherapy and radiotherapy provide the most established clinical platforms for assessing whether immune conditioning can improve responsiveness in biologically cold tumors. Both modalities are already part of standard care and can be evaluated in combination with checkpoint blockade. Their clinical relevance lies less in demonstrating complete biological conversion than in showing that treatment‑induced immune remodeling can improve outcomes in settings otherwise resistant to immunotherapy.

Among available approaches, chemo‑immunotherapy offers the most mature clinical support. In colorectal cancer, trifluridine/tipiracil combined with oxaliplatin triggered ICD through eIF2α phosphorylation, enhanced the efficacy of PD‑1 blockade, depleted suppressive macrophages and increased T‐cell infiltration [259]. In NSCLC, platinum‑ and taxane‑containing regimens have been associated with immunogenic‑cell‑death‑related immune activation [260, 261]. These observations align with the Phase III KEYNOTE‑189 and IMpower132 trials, in which PD‑1 or PD‑L1 inhibitors combined with chemotherapy prolonged overall survival relative to chemotherapy alone [171, 172]. Similar immune‑conditioning effects have been suggested in gastric cancer and hepatocellular carcinoma, where chemotherapy may enhance dendritic‐cell activation, reduce metabolic immunosuppression, and synergize with checkpoint blockade [257, 262]. However, these studies do not establish complete cold‐to‐hot conversion in prospectively stratified cold tumor populations.

Radiotherapy exerts immune‐enhancing effects that are highly context dependent. In the Phase III PACIFIC trial, durvalumab consolidation after chemoradiotherapy improved outcomes in unresectable NSCLC [263, 264], supporting the concept that radiotherapy may provide immune priming within an appropriate treatment context. Radiotherapy has also increased T‐cell infiltration and induced abscopal‐like effects in hepatocellular carcinoma with portal vein thrombosis and in melanoma brain metastases [253, 265, 266]. Other local strategies, including photodynamic therapy combined with anti‐PD‐1 agents, may similarly promote antigen release and innate immune activation [267]. At present, durable clinical benefit is best supported for integrated chemoradiotherapy or radio‐immunotherapy regimens rather than for local immune conditioning used as a stand‐alone intervention.

#### 6.1.2 Emerging Priming‐Oriented Strategies

These emerging strategies parallel chemotherapy and radiotherapy in restoring tumor immune visibility, but clinical evidence remains preliminary and durable conversion uncertain. To date, they have shown immune activity without clear evidence of sustained phenotypic conversion. T‑VEC achieved a durable response rate of 16.3% in advanced melanoma, with synergistic activity reported in combination with pembrolizumab [130]. Personalized mRNA vaccines, including mRNA‐4157 and BNT111, have increased immunogenicity in melanoma [169], whereas autogene cevumeran has shown early clinical activity in pancreatic cancer [198, 268]. Epigenetic modulators such as azacitidine can reverse immune gene silencing, increasing tumor visibility, and lymphocyte recruitment [7, 269, 270]. Their application is constrained by schedule dependence, prior treatment exposure and persistent microenvironmental suppression. Clinical evidence supports the feasibility of increasing tumor immunogenicity, but whether such changes produce durable reversal of cold tumor biology remains unresolved.

### 6.2 Clinical Relief of Exclusion and Restoration of Immune Access

Unlike priming failure, immune exclusion involves physical barriers that block activated immune cells from entering tumors; vascular and stromal remodeling are the most direct strategies to address it. Interventions targeting these barriers test whether structural restriction can be therapeutically modified without unacceptable toxicity.

Among microenvironment‑directed strategies, vascular remodeling provides the most convincing evidence. Antiangiogenic agents such as bevacizumab have reduced hypoxia and improved T‐cell penetration in hepatocellular carcinoma and renal cell carcinoma [220, 258], whereas combinations such as avelumab plus axitinib have reshaped the tumor microenvironment and produced durable disease control in renal cell carcinoma [183]. Stromal modulation has also shown preliminary activity. LOXL2 inhibition has alleviated stromal restriction in pancreatic ductal adenocarcinoma, and TGF‑β inhibition has reduced cancer‑associated fibroblast activation in triple‑negative breast cancer [271, 272, 273]. These studies support the clinical modifiability of immune exclusion, particularly in immune‐excluded tumors.

### 6.3 Clinical Preservation or Restoration of Effector Fitness

Clinical strategies targeting effector function address tumors in which immune cells are present or can be supplied, but remain functionally constrained. The central question is whether cytotoxic lymphocytes can be sustained long enough to achieve durable antitumor control.

#### 6.3.1 Checkpoint Blockade in Biologically Constrained Tumors

Checkpoint blockade directly targets pre‑existing but dysfunctional immune responses; its limited activity in strongly cold tumors underscores that effector restoration alone is insufficient when upstream defects in priming or access remain uncorrected. Dual‐checkpoint regimens have shown superior efficacy in melanoma and NSCLC, whereas checkpoint monotherapy has been markedly less effective in glioblastoma [274, 275].

#### 6.3.2 Metabolic and Other Context‐Dependent Approaches

Metabolic interventions have yielded inconsistent clinical signals, complicated by intertumoral heterogeneity, compartment‑specific pharmacology and uncertainty about whether the targeted pathway is rate limiting in the treated population. Inhibitors of glutamine, adenosine or glycolytic pathways have been developed to alleviate metabolic suppression, but durable efficacy has generally been limited [8, 184, 276, 277, 278]. Ferroptosis‑ and cuproptosis‑directed strategies conceptually link metabolic damage to immune activation, but remain early in development [7, 279, 280, 281]. These findings indicate that preserving immune fitness is clinically relevant but difficult to achieve selectively and durably.

#### 6.3.3 Cellular Therapies and Unresolved Access Constraints

Cellular therapies directly supply tumor‑reactive effector cells. Their performance in solid tumors is particularly informative because it reveals the extent to which trafficking barriers, intratumoral hostility and limited persistence continue to constrain highly engineered interventions. CAR‑T cells have achieved transformative efficacy in hematological malignancies, but their activity in solid tumors has been limited by impaired trafficking, insufficient persistence and hostile intratumoral conditions [282, 283]. Newer approaches, including γδ T cells and cytokine‑armed CAR‑T cells, remain under investigation [284, 285, 286]. These findings indicate that cellular delivery alone does not overcome the full architecture of cold tumor disease. Effector restoration is an essential component of cold‑to‑hot conversion, but is most effective in tumors with at least partial baseline immune engagement or after prior correction of upstream barriers.

### 6.4 Clinical Reprogramming of Suppressive TME States

Clinical strategies for reprogramming suppressive microenvironmental states are less mature than priming‑ or access‑oriented approaches. Microbiota‑directed interventions and therapies targeting suppressive immune populations have generated context‑dependent signals, but consistent validation across tumor types and treatment settings remains lacking [287, 288, 289]. This limited success does not invalidate the underlying biology. Rather, it highlights a recurring translational problem: in complex human tumors, clinical failure may reflect inadequate identification of the dominant barrier, insufficient intratumoral target engagement, compensatory suppressive pathways or inappropriate patient selection.

The IDO1 experience illustrates this challenge. Despite strong preclinical rationale, late‑stage trials failed to deliver expected benefit, likely because tryptophan metabolism was not uniformly dominant across enrolled tumors and could be buffered by TDO and parallel immunosuppressive circuits. Pharmacodynamic markers of pathway dependence were limited, and patients were not selected using a validated biomarker framework capable of identifying truly IDO‑dependent lesions [290]. Suppressive TME reprogramming therefore cannot be advanced on mechanistic plausibility alone; successful translation will require prospective evidence of pathway dominance, adequate target engagement at the tumor site and biomarker‑defined patient selection.

### 6.5 What Current Clinical Translation Establishes for Barrier‐Matched Combinations

Current clinical evidence establishes cold‑to‑hot tumor conversion as a translatable therapeutic concept, not merely a preclinical construct. Chemo‑immunotherapy and chemoradiotherapy are now part of standard care in multiple tumor types [119, 171, 172, 256, 257], and antiangiogenic combinations have provided clinical evidence that immune exclusion can be alleviated [183, 220, 258]. Efficacy is also strongly sequence‑dependent. In biologically cold settings, tumor priming before checkpoint blockade generally outperforms checkpoint monotherapy, and neoadjuvant or perioperative contexts may be particularly favorable because earlier‑stage tumors often retain more intact antigen sources and less entrenched immune escape [119, 291].

Current evidence, however, falls short of defining cold‑to‑hot conversion as a standardized therapeutic strategy. Most trials have not prospectively stratified patients according to barrier architecture, and few have linked serial biological measurements to durable patient benefit. Cold‑to‑hot conversion is therefore a clinically relevant objective, but not yet a standardized strategy. Its implementation will require a more precise, barrier‑defined, and mechanism‑based translational framework. Representative ongoing and clinically relevant trials, including NCT numbers, trial status, phase, therapeutic objectives, and preliminary findings, are summarized in Table 1.

## 7 Biomarkers and Predictive Models for Cold‐to‐Hot Tumor Conversion

A major barrier to clinical implementation is that cold tumors do not constitute a single biological entity. In some lesions, the dominant defect is failed antigen priming; in others, immune exclusion, suppressive myeloid circuitry, or pre‑existing but functionally constrained immunity predominates. Biomarkers must therefore serve multiple roles: baseline stratification, on‑treatment monitoring of conversion, and identification of unresolved barriers that account for therapeutic failure.

**FIGURE 4:**
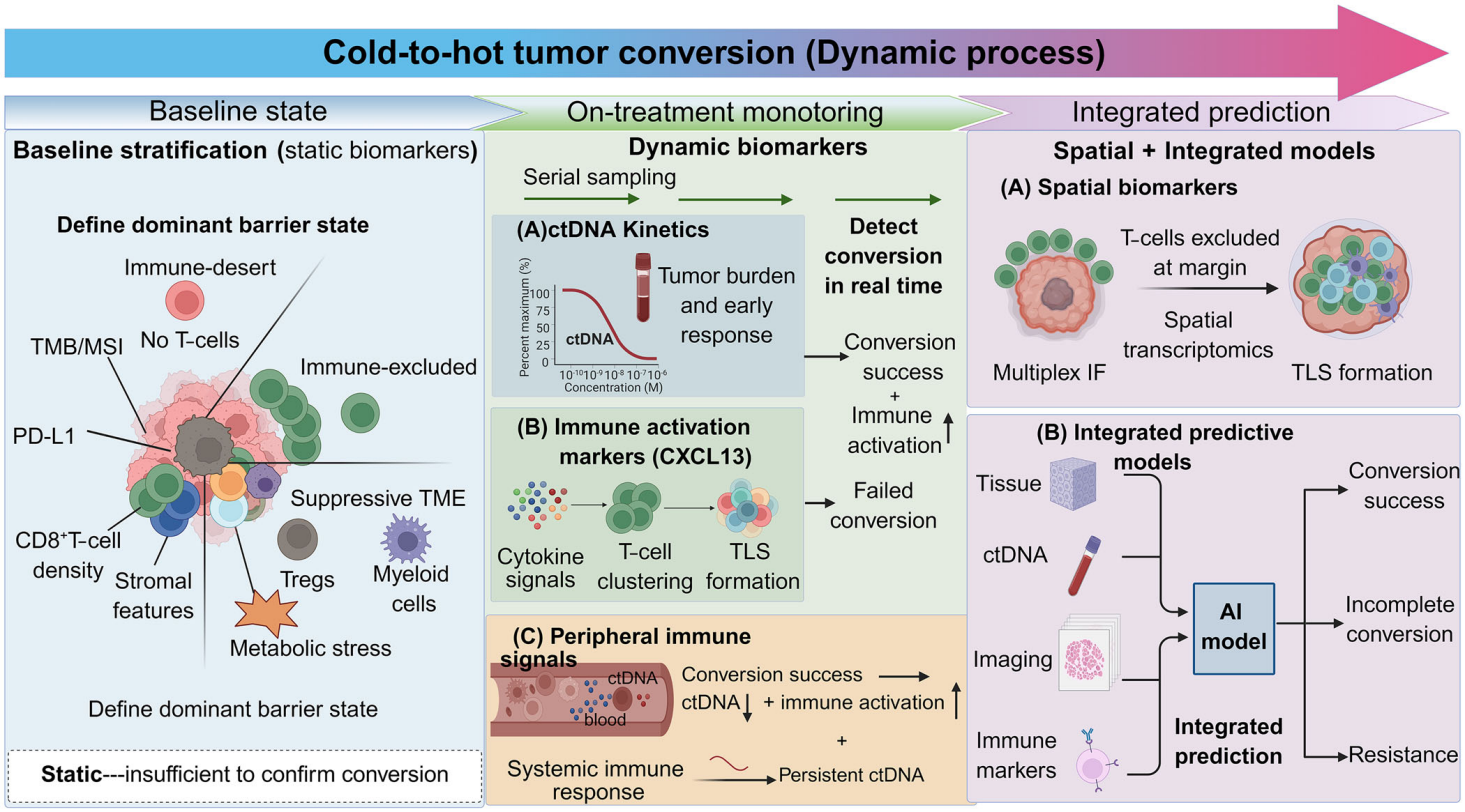
Biomarker‐guided monitoring and prediction of cold‐to‐hot tumor conversion. The figure outlines a biomarker framework for assessing cold‐to‐hot tumor conversion as a dynamic process. Baseline stratification defines dominant barrier states using static biomarkers, including TMB/MSI, PD‐L1 expression, CD8^+^ T‐cell density, stromal features, suppressive immune cells, and metabolic stress. On‐treatment monitoring incorporates dynamic biomarkers, including ctDNA kinetics, immune activation markers such as CXCL13 and peripheral immune signals, to track tumor burden, immune activation and conversion status. Spatial biomarkers, including multiplex immunofluorescence and spatial transcriptomics, assess immune‐cell localization, tumor‐core infiltration, and TLS formation. Integrated predictive models combine tissue, ctDNA, imaging and immune‐marker data to classify conversion success, incomplete conversion or resistance. ctDNA, circulating tumor DNA; IF, immunofluorescence; MSI, microsatellite instability; TLS, tertiary lymphoid structure; TMB, tumor mutational burden; TME, tumor microenvironment.

This has important implications for biomarker design. Static markers remain useful for defining pretreatment context and enriching trial populations, but they cannot capture the transient, treatment‑dependent biology of conversion. Dynamic markers are better suited to tracking real‑time microenvironmental remodeling, immune activation, and incomplete or abortive conversion. Predictive models should therefore integrate baseline barrier state, on‑treatment kinetics and selected spatial information, rather than rely on any single pretreatment variable [292]. A more comprehensive framework that integrates static, dynamic, and spatial biomarkers for monitoring conversion is outlined in Figure 4. Representative biomarker categories and their translational uses are summarized in Table 2.

**TABLE 2:** Biomarkers for baseline stratification, on‐treatment conversion monitoring, spatial architecture assessment, and integrated prediction in cold‐to‐hot tumor conversion.

Clinical biomarker category	Representative examples	Sample source	Platform	Most informative	Primary translational use	Main limitation	Refs.
Baseline immunogenicity and priming markers	TMB; MSI‐H/dMMR; antigen‐presentation defects; interferon‐pathway alterations	Tumor tissue	Genomic profiling; targeted sequencing; panel‐based assays	Priming‐deficient states and likely baseline immunogenicity	Baseline stratification for likely responsiveness to priming‐oriented or checkpoint‐based therapy	Limited specificity for dominant barrier state; substantial intertumoral heterogeneity	[293, 294, 295, 296]
Baseline immune‐state and suppressive‐TME markers	IFN‐γ‐related signatures; chemokine signatures (CXCL9/CXCL10/CXCL11); stromal exclusion patterns; CD8 density; PD‐L1; suppressive myeloid or regulatory‐cell enrichment	Tumor tissue	RNA‐seq; IHC; multiplex IF; digital pathology	Noninflamed versus inflamed states; exclusion‐dominant or suppressive‐TME states	Baseline assignment of likely dominant barrier state	Platform variability; assay‐dependent interpretation; incomplete transferability across tumor types	[26, 297]
On‐treatment dynamic conversion markers	ctDNA decline; peripheral immune‐cell subsets; soluble immune mediators; selected immune activation signals such as CXCL13	Plasma; blood; selected paired tissue samples	Liquid biopsy; flow cytometry; multiplex cytokine assays; serial tissue profiling	Early biological response, treatment‐associated immune activation, and possible conversion during therapy	On‐treatment monitoring of whether biological remodeling is occurring	Does not directly resolve intratumoral spatial redistribution; thresholds are not standardized across indications	[298, 299, 300]
Spatial architecture biomarkers	Spatial exclusion patterns; immune‐cell redistribution; local checkpoint topology; stromal–immune compartmentalization	Tumor tissue	Multiplex IF; spatial transcriptomics; image‐based spatial profiling	Exclusion mapping and local barrier architecture	Determination of whether immune cells are absent, margin‐restricted, or redistributed after treatment	Limited standardization; cost; restricted scalability for routine clinical use	[301, 302, 303]
Noninvasive imaging biomarkers	CT‐ and PET‐derived radiomic features; delta‐radiomics patterns	Imaging	Radiomics; delta‐radiomics; model‐based imaging analysis	Longitudinal response assessment and possible exclusion‐dominant states	Noninvasive tracking of treatment response and distinction of pseudoprogression from true progression in selected settings	Model robustness, external validation, and biological interpretability remain variable	[304, 305, 306]
Integrated predictive models	Multiomics classifiers; AI pathology models; histomorphology‐plus‐clinical models	Tissue; blood; routine pathology; clinical data	Multiomics integration; machine learning; AI‐assisted pathology	Complex, mixed, or heterogeneous barrier states	Hypothesis‐generating support for treatment assignment and prognostic prediction	Requires prospective external validation and standardized implementation pipelines; current clinical utility remains limited	[307, 308]

Abbreviations: AI, artificial intelligence; CT, computed tomography; ctDNA, circulating tumor DNA; dMMR, mismatch repair deficiency; IF, immunofluorescence; IFN, interferon; IHC, immunohistochemistry; MSI‐H, microsatellite instability‐high; PET, positron emission tomography; RNA‐seq, RNA sequencing; scRNA‐seq, single‐cell RNA sequencing; TMB, tumor mutational burden; TME, tumor microenvironment.

### 7.1 Baseline Biomarkers: Necessary for Stratification, Insufficient for Documenting Conversion

Pretreatment biomarkers remain necessary for estimating the dominant barrier architecture in a given tumor. Genomic variables such as tumor mutational burden (TMB) and microsatellite instability (MSI) reflect the potential for neoantigen generation and immune recognition, and retain value for identifying tumors with greater baseline immunogenic potential [298, 309, 310]. PD‑L1 expression similarly retains practical relevance in clinical decision‑making, particularly in the context of checkpoint blockade, despite well‑recognized limitations in assay heterogeneity, dynamic regulation, and intratumoral spatial variation [311, 312, 313].

Transcriptomic and histomorphological features can further refine baseline classification. Inflamed tumors are often enriched for interferon‑responsive genes, chemokines associated with T‐cell recruitment and markers of dendritic‐cell activation, whereas cold tumors more frequently exhibit impaired antigen‑presentation programs, stromal signatures, suppressive myeloid features, and exclusion‑associated extracellular matrix pathways [314, 315]. CD8^+^ T‐cell density, stromal fibrosis and immune cell localization patterns further distinguish immune‑desert from immune‑excluded phenotypes [316, 317].

These markers, however, have clear limitations. TMB, MSI, PD‑L1, and related baseline variables can support stratification, but they cannot capture the dynamic remodeling that defines successful conversion. A tumor with low baseline inflammation may become immunologically engaged under treatment, whereas a tumor with apparently favorable pretreatment features may fail to convert if immune exclusion, myeloid suppression or metabolic hostility persist. Baseline markers should therefore be viewed as stratification tools, not as direct indicators of conversion itself.

### 7.2 Dynamic Biomarkers: Monitoring Conversion in Real‐Time

The central challenge is to detect conversion as an active biological process, not to infer it retrospectively from radiographic response or survival. This places a premium on serial, minimally invasive and functionally interpretable markers.

Among currently available approaches, circulating tumor DNA (ctDNA) is the most clinically actionable dynamic marker. A serial decline after treatment can provide early evidence of effective tumor debulking and, in some settings, successful microenvironmental remodeling, often preceding radiographic response [318, 319]. In the context of cold‑to‑hot conversion, its value lies less in monitoring tumor burden alone than in offering a kinetic readout that can be aligned with immune activation and treatment sequencing. Persistent ctDNA despite treatment may indicate failed conversion, inadequate target engagement or early adaptive resistance. ctDNA should therefore be viewed as a core element of dynamic conversion monitoring, not simply as a response biomarker.

Markers of immune activation are equally important, because tumor shrinkage alone does not establish bona fide biological conversion. CXCL13 is particularly noteworthy in this regard. It is associated with intratumoral immune activation, lymphoid cell recruitment and tertiary lymphoid structure formation, and its induction during therapy may signal the emergence of a more permissive immune state [320, 321]. Its value is greatest when assessed longitudinally, as it may capture the transition point at which a previously cold lesion begins to acquire local immune organization and productive effector engagement.

Other dynamic biomarkers may provide complementary information. Peripheral immune cell subsets, soluble interferon‑related mediators, and treatment‑induced changes in inflammatory chemokines can help distinguish productive immune activation from nonproductive inflammation [322, 323]. Imaging‑based approaches, including radiomics and delta‑radiomics, may also contribute temporal information by capturing treatment‑induced changes in tissue texture, vascularity, and immune‑associated imaging features [324, 325, 326]. Even so, liquid biopsy‐based approaches are more readily scalable for repeated monitoring, particularly when the timing of conversion is uncertain and serial sampling is required.

Collectively, these considerations argue for a conceptual shift in biomarker strategy. The most informative biomarkers for cold‑to‑hot conversion are unlikely to be those that define baseline tumor class alone, but those that register whether a biologically cold lesion is actively transitioning under treatment. ctDNA kinetics and inducible activation markers such as CXCL13 are therefore of particular importance, because they can capture the timing, magnitude and incompleteness of conversion in ways that static tissue markers cannot.

### 7.3 Spatial Biomarkers and Integrative Predictive Models

Spatial biomarkers are conceptually important because cold‑to‑hot conversion is, in part, a spatial event. Distinguishing immune‑desert from immune‑excluded tumors, redistributing effector cells into tumor nests and assembling tertiary lymphoid structures all demand spatially resolved assessment. Multiplex immunofluorescence, spatial transcriptomics, and integrated platforms such as AstroPath can provide rich descriptions of tissue architecture, immune localization, and cell–cell organization [287, 323, 327, 328]. In principle, these technologies can determine whether immune cells remain margin‑restricted, whether stromal barriers are being relaxed and whether local immune niches are being reassembled during therapy.

Their translational limitations, however, warrant equal attention. Although spatial platforms generate biologically informative data, routine clinical deployment is constrained by preanalytical variability, platform‑specific workflows, limited cross‑site standardization, computational complexity, prolonged turnaround time, and high cost. AstroPath and related approaches are therefore best viewed, at present, as powerful translational research tools rather than readily scalable clinical assays [329]. Their current strength lies in mechanistic resolution, not in routine implementation.

For this reason, future predictive models will likely depend on integration across platforms. The most useful frameworks will combine baseline stratification, serial dynamic monitoring, and selected spatial features when clinically feasible. Machine learning may help by integrating histomorphology, radiographic change, ctDNA kinetics, immune activation markers, and transcriptomic context into unified predictive models [329, 330]. The goal is not simply to build increasingly complex systems, but to generate clinically usable frameworks that identify the dominant barrier at baseline, detect whether conversion is occurring during treatment and clarify why it fails when benefit is absent.

Biomarkers and predictive models are therefore indispensable, not only for patient selection, but also for redefining cold‑to‑hot conversion as a measurable and dynamically testable process. Future progress will depend less on refining static classifiers than on standardizing longitudinal liquid‑biopsy workflows, validating on‑treatment activation markers such as CXCL13, and defining the spatial resolution required for clinical decisions rather than for biological description alone.

## 8 Safety and Toxicity Considerations for Cold‐to‐Hot Tumor Conversion

Safety is a central determinant of whether cold‐to‐hot tumor conversion can be clinically deployed. As summarized in Table 3, the major toxicities associated with conversion strategies do not arise from a single treatment class, but from the same biological processes that are intended to restore antitumor immunity. PD‐1/PD‐L1 blockade provides the current safety benchmark, whereas CTLA‐4‐containing or dual‐checkpoint regimens narrow the therapeutic window through broader immune activation. When immune checkpoint inhibitors are combined with chemotherapy, radiotherapy or antiangiogenic agents, immune release is superimposed on tissue injury, myelosuppression, endothelial stress or vascular remodeling. Similarly, epigenetic sensitizers, metabolic modulators, innate immune agonists, oncolytic viruses, and ferroptosis‐inducing strategies may promote tumor immune activation, but they also expose normal proliferative, immune, vascular or epithelial compartments to overlapping inflammatory and metabolic stress.

**TABLE 3:** Safety constraints across clinically relevant and emerging cold‐to‐hot tumor conversion strategies.

Strategy	Representative intervention	Clinical trial or evidence status	Study purpose	Reported signal	Main safety issue	Refs.
ICI toxicity benchmark/ICI	Anti‐PD‐1/PD‐L1 agents, including nivolumab, pembrolizumab, atezolizumab	Clinical safety synthesis; multiple trials	Define baseline toxicity of immune activation	irAEs may involve skin, gastrointestinal tract, liver, lung, endocrine organs and blood system	Multiorgan irAEs; delayed onset and difficult attribution	[331, 332, 333, 334]
PD‐1 + CTLA‐4 dual blockade/PD‐1 + CTLA‐4	Nivolumab + ipilimumab	NCT01844505; Phase III; results reported	Evaluate long‐term efficacy and safety in advanced melanoma	Median OS: 72.1, 36.9 and 19.9 months for combination, nivolumab and ipilimumab	High‐grade irAEs, especially colitis, hepatitis and endocrinopathy	[335]
PD‐1 + LAG‐3 dual blockade/PD‐1 + LAG‐3	Relatlimab + nivolumab	NCT03470922; Phase 2–3; results reported	Compare LAG‐3/PD‐1 blockade with PD‐1 blockade alone	Median PFS: 10.1 versus 4.6 months; grade 3/4 TRAEs: 18.9% versus 9.7%	Increased immune toxicity; liver enzymes, thyroiditis and pneumonitis require monitoring	[187]
Postoperative ICI + CRT	Nivolumab + postoperative RT/cisplatin	NCT03576417; Phase III; ongoing with reported conference data	Evaluate adjuvant nivolumab in high‐risk resected	DFS improvement without compromising compliance or surgical feasibility	Additive radiation injury and irAEs	[336, 337]
ICI + definitive CRT/ICI	Avelumab + CRT	NCT02952586; Phase III; terminated; results reported	Test PFS benefit in LA‐HNSCC	Primary PFS endpoint not met; no OS benefit	Narrow therapeutic window due to CRT toxicity	[336, 338]
ICI + concurrent CRT/ICI	Pembrolizumab + CRT	NCT03040999; Phase III; results reported	Test EFS benefit in LA‐HNSCC	Primary EFS endpoint not met; numerical trend favored pembrolizumab	Added toxicity without clear biomarker‐defined benefit	[336, 339]
HPV vaccine + PD‐1 blockade	PDS0101 + pembrolizumab	NCT04260126; Phase II; ongoing	Evaluate safety and activity in HPV16+ HNSCC	HPV‐specific immune responses and durable activity in a subset	Immune activation and PD‐1‐related toxicity	[336]
Listeria‐based HPV immunotherapy	ADXS11‐001 ± durvalumab	NCT02002182; Phase I/II; completed	Evaluate feasibility and immunogenicity in HPV‐associated cancers	Feasible and tolerable; robust HPV‐specific CD8+ responses	Live‐vector infection risk and inflammation	[336, 340]
HPV vaccine platform + PD‐L1 blockade/HPV PD‐L1	MEDI0457 + durvalumab	NCT03439085; Phase II; active, not recruiting	Explore activity across HPV16/18‐associated cancers	Consolidated results NR	Tumor‐type‐specific safety and efficacy uncertainty	[336]
Microbiome modulation + ICI	Oral inulin + nivolumab or pembrolizumab ± chemotherapy	NCT05821751; phase NR; ongoing	Assess microbiome and immune changes in R/M‐HNSCC	Interim: 9/16 patients achieved PFS ≥ 6 months	Heterogeneous microbiome response; limited safety data	[336, 341]
Salivary microbiome biomarker	Pretreatment saliva metagenomics	NCT04649476; phase/status	Predict neoadjuvant ICI response in oral SCC	Lower microbial diversity in nonresponders; AUC = 0.81	Biomarker validation, not direct therapeutic toxicity	[336, 342]
Glucose restriction sensitization	FMD + 2‐DG ± anti‐PD‐1/PD‐L1; EDEM3–PD‐L1 glycosylation axis	preclinical/translational	Reverse PD‐L1 glycosylation and M2‐like macrophage recruitment	Increased CD8+ T‐cell infiltration and reduced M2‐like macrophages	Nutritional/metabolic stress and impaired normal immunity	[343]
Epigenetic/IDO‐related modulation + ICI	Azacitidine or INCB057643/INCB059872 + pembrolizumab + epacadostat	NCT02959437; phase/status NR	Test epigenetic or IDO‐associated sensitization across solid tumors	Results NR	Hematologic, metabolic and immune toxicity attribution	[344]
Antiangiogenic ICI combination	ICI + antiangiogenesis + chemotherapy	NCT NR in cited PDF; clinical evidence synthesis	Evaluate ICI‐based therapy after EGFR‐TKI resistance in EGFR‐mutated NSCLC	ICI‐antiangio‐chemo showed best PFS/ORR/DCR ranking but higher AE ranking	Hematologic toxicity, vascular toxicity and cumulative AEs	[345]
Glutamine metabolism modulation	JHU083/DON prodrug ± anti‐PD‐1/anti‐CTLA‐4	preclinical	Remodel suppressive myeloid cells and sensitize ICI‐resistant tumors	Reduced MDSCs, increased inflammatory TAMs and CD8+ T‐cell response; enhanced checkpoint blockade	Systemic metabolic toxicity and possible effector‐cell impairment	[346]
STING agonist ADC	Tumor‐cell‐directed STINGa ADC	preclinical	Deliver STING agonist to tumor and myeloid cells	Activated STING and Type III IFNs; lower serum cytokine elevation than free	Cytokine toxicity and off‐tumor innate immune activation	[347]
Oncolytic virotherapy + ICI	T‐VEC, HF10, CAVATAK, ONCOS‐102, Enadenotucirev, Pexa‐Vec, VSV‐IFNβ‐NIS and other OV platforms	Multiple NCTs summarized; phase/status variable	Use viral lysis and ICD to convert cold tumors to hot tumors	Some combinations reported ORR signals, but efficacy varies /	Unique issues: Poor intratumoral spread, host antiviral immunity, viral clearance, route‐dependent inflammation and patient selection	[348]
Ferroptosis–autophagy–immunity platform	Injectable SF‐HA/Fe_3_O_4_/sorafenib hydrogel + AMF + TAT‐Beclin1	preclinical	Induce ferroptosis, ICD and CD8+ T‐cell activation in TNBC	Local ROS generation, GPX4 suppression and immune activation; no systemic toxicity reported in preclinical testing	Local hyperthermia, ROS injury and iron/Fenton toxicity	[349]
Ferroptosis therapeutic framework	GPX4–system Xc−–lipid peroxidation axis	Mechanistic review; no clinical	Define ferroptosis as a tumor vulnerability and immune‐related mechanism	Therapy‐resistant cancer cells may be vulnerable to ferroptosis	Off‐tumor lipid peroxidation and organ injury	[350]
Fe^2^^+^ metabolism modulation	DHU‐Feex1 Fe^2^^+^ metabolism modulator	preclinical nontumor immune‐stress model	Regulate Fe^2^^+^‐driven ferroptosis and inflammation	Prolonged graft survival, reduced rejection and inflammation	Iron‐homeostasis disturbance and oversuppression of immune stress	[351]

Abbreviations: ADC, antibody–drug conjugate; BORR, best overall response rate; CRC, colorectal cancer; CRT, concurrent chemoradiotherapy; DFS, disease‐free survival; DCR, disease control rate; EAS, ecological association score; EFS, event‐free survival; FMD, fasting‐mimicking diet; HCC, hepatocellular carcinoma; HNSCC, head and neck squamous cell carcinoma; ICD, immunogenic cell death; ICI, immune checkpoint inhibitor; irAE, immune‐related adverse event; LA‐HNSCC, locally advanced head and neck squamous cell carcinoma; MDSC, myeloid‐derived suppressor cell; MSS CRC, microsatellite stable colorectal cancer; NR, not reported; NSCLC, non—small‐cell lung cancer; ORR, objective response rate; OS, overall survival; PD‐L1, programmed death‐ligand 1; PFS, progression‐free survival; R/M‐HNSCC, recurrent/metastatic head and neck squamous cell carcinoma; RT, radiotherapy; SCC, squamous cell carcinoma; TAM, tumor‐associated macrophage; TNBC, triple‐negative breast cancer; TRAE, treatment‐related adverse event.

The safety challenge in cold‐to‐hot conversion is therefore structural rather than merely descriptive. Listing adverse‐event frequencies is insufficient, because efficacy and toxicity often reflect partially shared mechanisms: antigen release, innate immune activation, checkpoint release, effector‐cell recruitment, stromal or vascular remodeling, and oxidative or metabolic perturbation. These processes must be strong enough to overcome immune ignorance, exclusion, or suppression, but sufficiently restricted to avoid systemic inflammation and off‐tumor tissue injury. The clinically relevant question is thus not only which agent produces toxicity, but which route, sequence, dose intensity, exposure profile, and patient context can preserve tumor‐directed immune activation while maintaining an acceptable therapeutic window.

### 8.1 Therapeutic‐Window Compression as the Organizing Principle

Therapeutic‑window compression helps to conceptualize safety in cold‑to‑hot conversion [352, 353]. Safety is not a secondary concern to be addressed after efficacy has been established; it is a primary determinant of clinical viability.

This compression is most pronounced in multimodal regimens. Checkpoint blockade releases pre‑existing immune restraint; chemotherapy and radiotherapy increase both antigen availability and tissue injury; and microenvironmental modulators alter vascular, stromal, or metabolic barriers. When combined, these effects are not merely additive. Tissue injury, inflammatory amplification, and immune disinhibition reinforce one another and further narrow the interval between ineffective treatment and unacceptable toxicity. Regimens may therefore fail not simply because they lack biological potency, but because the immunological intensity required for conversion cannot be confined in space, duration, or systemic exposure [354].

### 8.2 Immune‐Related Toxicity and Efficacy Are Mechanistically Coupled

Immune‑related adverse events (irAEs) are often framed as complications of successful immunotherapy. In the context of cold‑to‑hot conversion, however, they are more accurately viewed as manifestations of the same immune escalation that may also drive tumor control. Clinical associations between irAEs and improved outcomes are therefore biologically plausible, though neither universal nor sufficient to establish causality [355, 356, 357, 358, 359]. In many cases, both efficacy and toxicity appear to arise from shared processes: broadened T‐cell activation, T‐cell receptor clonotype expansion, epitope spreading, cytokine amplification, and disruption of inhibitory circuits that normally restrain immune reactivity.

This coupling is most evident in checkpoint inhibitor‑based regimens. PD‑1/PD‑L1 blockade restores exhausted effector function, whereas CTLA‑4 blockade more broadly perturbs peripheral tolerance and regulatory T‐cell control [360]. Dual‐checkpoint blockade, or checkpoint inhibition combined with chemotherapy, radiotherapy or antiangiogenic agents, can intensify immune activation while simultaneously increasing tissue injury and antigen release [361, 362]. Under these conditions, tumor rejection and irAEs may represent divergent outcomes of a shared immunological process rather than separate biological events.

That conclusion nonetheless warrants methodological caution. Associations between irAEs and improved outcomes should not be interpreted uncritically, as they may be influenced by treatment exposure, time‑on‑therapy effects, disease‑specific context, and related biases [363]. The relevant implication is not that toxicity should serve as a surrogate for efficacy, nor that greater toxicity is desirable. Rather, the field must move beyond the assumption that efficacy and toxicity can be independently optimized through empirical dose adjustment alone. In cold‑to‑hot conversion, the central challenge is how to preserve the biological processes required for productive antitumor immunity while limiting their systemic spillover and temporal persistence.

### 8.3 How Safety Compression Manifests Across Conversion Strategies

Across conversion strategies, toxicity intensifies when the biological processes required for local immune activation are not confined to the tumor. The common safety logic across otherwise distinct regimens is therefore loss of tumor restriction.

Checkpoint inhibitor‑based combinations provide the clearest clinical example. Compared with PD‑1/PD‑L1 blockade alone, CTLA‑4 inhibition or dual‐checkpoint blockade is associated with substantially higher rates of severe irAEs [148, 335, 355, 356, 357, 364]. This pattern indicates that broader systemic immune disinhibition narrows the usable therapeutic range. The same logic extends to combinations with chemotherapy, radiotherapy, or antiangiogenic agents, where immune release is superimposed on tissue injury, myelosuppression, endothelial stress, and organ‑specific inflammatory vulnerability [171, 215, 245, 365, 366, 367, 368, 369, 370, 371, 372, 373].

TME modulators illustrate a related but distinct form of safety compression. Epigenetic therapies, metabolic regulators, and innate immune agonists can restore tumor visibility, weaken suppressive barriers, or induce inflammatory remodeling, yet the pathways they target are often shared by normal proliferative tissues, hematopoietic compartments, endothelial cells, and immune cells. Systemic STING agonism exemplifies this tension. Although the pathway is attractive for initiating local immune activation, systemic activation can induce interferon‑driven toxicity, including flu‑like syndromes, hypotension, hepatic injury, and broader inflammatory spillover [374, 375, 376, 377]. The same constraint applies to epigenetic and metabolic therapies, where the biological rationale is limited by the difficulty of restricting exposure to the tumor.

Cell‑death‑based conversion strategies further underscore this point. Ferroptosis‑inducing approaches can promote inflammatory remodeling and immune recruitment within tumors, yet the lipid peroxidation programs involved are not inherently tumor‑specific and may also damage epithelial, hematopoietic, or immune compartments [125, 378, 379, 380, 381]. The safety problem is therefore not simply that these agents produce toxicity, but that the molecular machinery required for tumor‑directed inflammatory conversion is not naturally confined to tumor tissue.

### 8.4 Decoupling Efficacy From Toxicity: Spatial Restriction and Conditional Activation

If the safety problem in cold‐to‐hot conversion arises from loss of tumor restriction, the more durable strategy is not empirical dose reduction alone, but decoupling efficacy from toxicity through spatial and biochemical control. Two design principles are especially important: Spatial restriction and conditional activation.

Spatial restriction confines pharmacological activity to the tumor or its local environment. Local delivery of STING agonists, oncolytic viruses, cytokine‑inducing agents or inflammatory scaffolds increases local pharmacodynamic intensity while reducing systemic exposure [375, 376]. This principle is particularly relevant in cold tumors, where the goal is to induce local immune transition within a noninflamed lesion. The more effectively treatment remains site‑confined, the greater the chance of maintaining immune activation while limiting cytokine‑driven systemic toxicity, endothelial injury, and distant organ inflammation.

Conditional activation relies on tumor‑enriched biological conditions rather than anatomy alone [382]. Tumor‑activated prodrugs, masked antibodies, conditional cytokines, protease‑cleavable agonists, and related designs remain inert in circulation and activate only under tumor‑associated conditions such as hypoxia, acidic pH, protease activity, oxidative stress, or lineage‑restricted enzymatic programs. Their value lies in ensuring that inflammatory potency is preferentially revealed where conversion is required and minimized where toxicity would be prohibitive [383].

Treatment sequence and patient selection further determine clinical tolerability. A regimen that is intolerable when given concurrently may become feasible when inflammatory priming, checkpoint release and microenvironmental remodeling are delivered in a staged manner [384]. Safety also depends on identifying which patients require high‑intensity conversion and which tumors do not justify the immunological cost. Barrier identification therefore matters not only for efficacy, but also for avoiding unnecessary overtreatment. Cold‑to‑hot conversion will become clinically sustainable only when efficacy and toxicity are effectively uncoupled through spatial targeting, conditional activation, and mechanism‑based design. Safety constraints across relevant conversion strategies are summarized in Table 3.

## 9 Future Directions and Emerging Therapies for Cold‐to‐Hot Tumor Conversion

The goal of cold‑to‑hot tumor conversion is to extend immunotherapy from intrinsically inflamed tumors to a broader range of malignancies. Achieving this will require more precise resistance‐state classification, biology‑aligned intervention design, and durable clinical translation. Although technologies and combination strategies are advancing rapidly, translation remains constrained by limited mechanistic resolution, inadequate patient stratification and inefficient delivery.

Future work should therefore focus on four interconnected priorities: deeper characterization of the tumor microenvironment, optimization of existing therapeutic platforms, rational design of barrier‐directed combinations, and clinically actionable personalization. Progress in these areas will be essential not only for improving efficacy, but also for reducing empirical treatment selection, controlling toxicity, and establishing cold‐to‐hot conversion as a practical component of precision oncology.

### 9.1 Decoding the Tumor Microenvironment to Guide Precision Intervention

Greater biological and spatial precision in resolving the tumor microenvironment represents a major future direction. Single‐cell multiomics now enables integrated analysis of DNA, RNA, and protein states at cellular resolution, revealing the molecular diversity of tumor cells, immune infiltrates, and stromal compartments within individual lesions [385]. These approaches have refined the identification of cellular populations linked to immune exclusion and dysfunction, including exhausted T cells, myeloid‐derived suppressor cells, tumor‐associated macrophages, and cancer‐associated fibroblasts. This level of resolution shifts therapeutic reasoning away from broad phenotypic labels toward tractable cellular and molecular bottlenecks.

Spatial profiling provides an additional dimension of insight by showing how location, tissue architecture, and cell–cell interactions shape immune states. Spatial multiomics has clarified that immune exclusion often reflects organized spatial restriction, stromal compartmentalization and localized suppressive signaling rather than simply low immune cell counts [386, 387]. Multiplex imaging platforms, including mass spectrometry imaging and CODEX, further extend this framework by resolving immune checkpoints, chemokine gradients, and metabolic regulators within tissue context. Together, these technologies can identify immune‐privileged niches that shelter tumor cells from immune attack and help explain why otherwise rational therapies fail in selected lesions [388, 389].

Other platforms are likely to strengthen this precision framework. High‐throughput sequencing defines the clonal diversity and temporal dynamics of tumor‐reactive T cells during treatment, improving understanding of resistance, persistence, and immune escape [390, 391]. Patient‐derived organoid systems, particularly when cocultured with autologous immune components, provide a tractable setting for functional testing of barrier‐matched interventions and personalized regimen design [392, 393]. Their broader utility will depend on whether they can be integrated with blood‐based and imaging‐derived data to build clinically practical predictive frameworks for treatment assignment and response monitoring [394, 395].

### 9.2 Optimization of Current Conversion Modalities

Many leading conversion modalities are constrained by translational bottlenecks that limit the consistency and durability of responses. Adoptive cell therapy offers a direct means of supplying tumor‐reactive effectors, but its efficacy in cold solid tumors is restricted by complex manufacturing, poor trafficking, limited persistence, and suppression within hostile microenvironments [396, 397]. Progress will require improved in vivo delivery, enhanced resistance to local immunosuppressive signals, and better biomarkers for identifying patients most likely to benefit [398, 399]. Acquired resistance, including antigen loss and adaptive immune evasion, remains a major obstacle and will need more flexible and durable cellular designs [400, 401]. Advances in lipid nanoparticle delivery, engineered resistance circuits, and real‐time response monitoring may help address these limitations [402, 403].

Oncolytic virotherapy faces a related set of challenges. Its appeal lies in combining local tumor lysis, in situ immune priming, and microenvironmental reprogramming, yet clinical translation is limited by variable viral engineering, inefficient systemic delivery and uneven efficacy in heterogeneous tumors [208]. CRISPR‐Cas9‐based engineering offers a route to improvement by enabling multifunctional viruses that carry immunostimulatory cytokines, therapeutic transgenes, or editing cassettes designed to enhance immune activation and viral persistence [404, 405]. Advances in capsid engineering, nanoparticle encapsulation and cell‐mediated delivery may improve tumor tropism and broaden applicability to difficult solid tumors [406, 407]. At the same time, regulatory complexity, manufacturing scalability and cost remain major barriers to wider implementation [408]. The future of oncolytic virotherapy will therefore depend as much on delivery and manufacturability as on biological innovation itself [394, 409].

### 9.3 Emerging Combination Therapies and Personalized Therapeutic Platforms

The most effective future strategies are unlikely to rely on any single intervention. Progress will probably come from rational combinations that address multiple nonredundant barriers while remaining sufficiently selective and tolerable for clinical use. Combining oncolytic virotherapy with immune checkpoint blockade may enhance immune infiltration, broaden local inflammation, and help reverse T‐cell dysfunction in poorly inflamed tumors [410, 411]. Similarly, combining oncolytic virotherapy with adoptive cell approaches may improve effector‐cell persistence and intratumoral access, addressing both upstream and downstream constraints on cold tumor immunity [412]. The key challenge will be to define dosing, sequence, and patient eligibility in a way that preserves synergy without producing unmanageable toxicity.

Epigenetic modulation represents another important avenue for future combination design. DNMT inhibitors, EZH2 inhibitors, and m^6^A‐related regulators can reverse transcriptional silencing of tumor antigens and immune‐relevant genes, potentially restoring both tumor visibility and treatment responsiveness in immune‐refractory lesions [123]. Better understanding of how epigenetic regulation intersects with immune cell differentiation, exhaustion and microenvironmental suppression should reveal new vulnerabilities and improve the design of epigenetic‐immunotherapy combinations [413, 414]. Other emerging platforms, including the combination of adoptive cell therapy with neuroimmune modulation or targeted metabolic intervention, may further enhance effector function and relieve suppressive metabolic programs within the tumor microenvironment [415, 416]. In parallel, CRISPR‐Cas9‐based engineering is likely to refine cellular therapies by enabling more precise programming of antigen recognition, resistance to suppressive signals, and compatibility with multimodal regimens [415, 417].

Taken together, the future of cold‐to‐hot tumor conversion will depend on the convergence of mechanistic resolution, therapeutic engineering, and clinically actionable personalization. The objective is not simply to intensify immune activation, but to develop treatment platforms that are biologically matched, dynamically adaptable and clinically sustainable. With this goal achieved, cold‑to‑hot conversion would become an organizing principle in precision immuno‑oncology, rather than a promising concept with inconsistent clinical application.

## 10 Conclusion and Perspectives

The cold–hot tumor distinction offers a useful framework for understanding why immunotherapy achieves durable benefit in some cancers but not others. Cold tumors are not a single biological entity; they represent a spectrum of barrier‑defined states shaped by defective priming, immune exclusion, suppressive microenvironmental circuits, and adaptive resistance. This framework has reoriented therapeutic strategy from nonspecific immune stimulation toward barrier‑matched immune reprogramming.

Cold‑to‑hot conversion, however, is not a binary endpoint. Transient immune activation is generally easier to induce than durable immune control. A lesion may acquire inflammatory features during treatment yet still fail to generate sustained benefit, because stromal restriction persists, adaptive resistance emerges, or toxicity curtails treatment before remodeling can consolidate. The central question, therefore, is no longer whether a cold tumor can be heated, but whether an immune‑active state can be established, sustained, and spatially confined in a manner that is both biologically effective and clinically tolerable.

The obstacles ahead are tightly interrelated. Stromal and architectural barriers must be dismantled with sufficient precision to restore immune access without compromising normal tissue integrity or exacerbating systemic toxicity. Adaptive resistance that follows partial or transient conversion must also be anticipated; antigen loss, compensatory checkpoint upregulation, myeloid reprogramming, and metabolic rewiring can rapidly erode newly acquired immune activity. Furthermore, progress cannot be properly evaluated without a standardized framework for assessing conversion that incorporates baseline barrier classification, on‑treatment biological monitoring and clinically meaningful criteria that distinguish transient inflammatory changes from genuine therapeutic remodeling.

The roadmap toward 2030 therefore depends less on expanding individual platforms in isolation than on integrating mechanistic biology, dynamic biomarkers, spatially resolved yet clinically scalable tissue analysis, sequence‑aware therapeutic design, and biomarker‑guided patient selection into a coherent translational framework. The most transformative progress will come not from mere intensification, but from precise barrier‑state matching, real‑time tracking of conversion, and effective local confinement of immune activation.

The long‑term objective should therefore be defined more rigorously than cold‑to‑hot conversion alone. Conversion is not the endpoint. The clinically meaningful goal is the establishment of a sustainable hot tumor microenvironment in which immune activation is not merely induced but maintained with sufficient spatial organization, effector persistence, and therapeutic tolerability to support durable tumor control. Whether this state proves achievable will determine whether cold‑to‑hot conversion remains a useful conceptual framework or becomes a durable organizing principle of precision immuno‑oncology.

## Author Contributions

F. C. conceived the project. X. G. and D. H. wrote the original draft and revised the manuscript. X. G. also edited and prepared the figures. J. W., X. D., and C. M. prepared and compiled the tables. All authors reviewed and approved the final version of the manuscript.

## Ethics Statement

The authors have nothing to report.

## Conflicts of Interest

The authors declare no conflicts of interest.

## Data Availability

The authors have nothing to report.
